# 5-Aminolevulinic acid activates the MdWRKY71-MdMADS1 module to enhance anthocyanin biosynthesis in apple

**DOI:** 10.1186/s43897-024-00127-x

**Published:** 2025-02-03

**Authors:** Liuzi Zhang, Huihui Tao, Jianting Zhang, Yuyan An, Liangju Wang

**Affiliations:** 1 College of Horticulture, Nanjing Agricultural University, Nanjing, 210095 China; 2 College of Life Sciences, Shaanxi Normal University, Xi’an, 710119 China

**Keywords:** 5-Aminolevulinic acid (ALA), Anthocyanin biosynthesis, Apple, MdWRK71-MdMADS1 module, Transcriptional regulation

## Abstract

5-Aminolevulinic acid (ALA), as a natural plant growth regulator, is well known for promoting red fruit coloring by enhancing anthocyanin accumulation. However, the underlying mechanisms remain elusive. In this study, we firstly demonstrated that ALA upregulates gene expression of the transcription factor MdMADS1, which in turn directly binds to and activates transcription of the key anthocyanin biosynthetic genes, *MdCHS* and *MdUFGT*. Then, we identified a novel WRKY transcription factor, MdWRKY71, that interacts with MdMADS1. Through gene manipulation, we revealed that MdWRKY71 plays a pivotal role in ALA-induced anthocyanin accumulation, highlighting its regulatory significance in this process. Further investigation unveiled that MdWRKY71 not only activates *MdMADS1* transcription but also enhances its transcriptional activation on its target genes, *MdCHS* and *MdUFGT*. Additionally, we discovered that MdWRKY71 independently binds to and activates the transcription of two other anthocyanin biosynthetic genes, *MdANS* and *MdDFR*. The protein–protein interaction between MdWRKY71 and MdMADS1 amplifies the transcriptional activation of these genes by MdWRKY71. These findings delineate a fine and complex regulatory framework where MdWRKY71 and MdMADS1 coordinately regulate anthocyanin biosynthesis in apples, providing new insights into the molecular control of fruit coloration and offering potential target genes for breeding aimed at enhancing fruit quality.

**Supplementary Information:**

The online version contains supplementary material available at 10.1186/s43897-024-00127-x.

## Core

ALA significantly promotes apple anthocyanin accumulation, but its mechanism is still unclear. Our results show that the WRKY71-MADS1 regulatory module plays an important role in ALA-induced anthocyanin biosynthesis.

## Gene & accession numbers

The accession numbers are: *MdMADS1* (MD17G1065400/AAC25922.1), *MdWRKY71* (MD10G1266400/XM_008385286.3), *MdCHS* (MD04G1003300), *MdCHI* (MD07G1186300), *MdF3H* (MD15G1246200), *MdDFR* (MD15G1024100), *MdANS* (MD06G1071600), *MdUFGT* (MD01G1234400), *MdGSTF12* (MD17G1272100), *MdMYB10* (MD09G1278400), *MdbHLH3* (MD11G1286900), *MdbHLH33* (MD07G1137500), *MdTTG1* (MD14G1031200), and *MdACTIN* (LOC103453508).

## Introduction

Anthocyanins, a class of flavonoids that impart hues of pink, red, blue, or purple, are ubiquitously found in various plant tissues such as flowers, fruits, and seeds. In the case of fruits like apple, grape, strawberry, litchi, and bayberry, which are prized for their vibrant red or purple colors, anthocyanins are the primary pigments that determine the visual appeal and, consequently, the perceived quality and commercial value of these fruits. Moreover, anthocyanins stand out as potent natural antioxidants with a myriad of health benefits. They are known to contribute to anti-aging effects, offer protection against cancer and cardiovascular diseases, and efficiently neutralize reactive oxygen species, underscoring their significance in promoting human health (Eberhardt et al. 2000; Bae et al. 2006; An et al. 2015, 2017). Given their multifaceted roles, research into the biosynthesis of anthocyanins in fruits has become a focal point in the field of secondary plant metabolism. It is not only pivotal for enhancing the intrinsic quality of fruits but also for bolstering their commercial competitiveness, making it one of the most vibrant and dynamic areas of study.

In all living organisms, the synthesis of tetrapyrrole (porphyrin) molecules is initiated from a ubiquitous precursor, 5-aminolevulinic acid (ALA). ALA's role transcends its function as a mere metabolic intermediate; it has been recognized as a naturally occurring, safe, and multifaceted plant growth regulator, particularly at low concentrations (Akram & Ashraf 2013; Wang et al. 2023). This dual functionality has garnered significant interest in the scientific community. One of ALA's most remarkable applications is its ability to enhance the red coloration of fruits, a phenomenon that has been extensively studied in a variety of fruits, including apple (Wang et al. 2004, 2006; Xie et al. 2013; Zheng et al. 2018; 2021; Fang et al. 2022; Zhang et al. 2024), grape (Watanabe et al. 2006), pear (Xiao et al. 2012), peach (Ye et al. 2017), litchi (Feng et al. 2015), and strawberry (Li et al. 2016). These investigations highlight the substantial potential of ALA in advancing the cultivation of high-quality fruits, offering a promising avenue for horticultural practices. Our laboratory has been dedicated to uncovering the mechanisms behind ALA's enhancement of fruit coloration and made some progress in this area. In apples, ALA enhances anthocyanin accumulation by activating the expression of biosynthetic genes and transcription factors that are crucial for the biosynthesis and transportation of anthocyanins (Xie et al. 2013; Fang et al. 2020). Feng et al. (2016) identified a transcription factor (TF) MdMADS1 through suppression subtractive hybridization (SSH) and proteomic analysis of apple peel treated by ALA. After overexpressing (OE-) and RNA interfering (RNAi-) *MdMADS1* in ‘Fuji’ apple calli, they proposed the gene is essential for ALA-induced anthocyanin accumulation. Zheng et al. (2021) found that two MYB TFs, MdMYB9 and MdMYB10, were involved in ALA-induced anthocyanin accumulation by activating *MdMATE8* expression to form a protein implicated in the vacuolar membrane transport of anthocyanins. Furthermore, MdMYB110a, which positively regulates anthocyanin accumulation in apple, has been shown to participate in ALA-enhanced anthocyanin accumulation by binding to the anthocyanin transporter gene *MdGSFT12* (Fang et al. 2023). Recently, other TFs, including MdERF78 and MdNAC33, have also been reported to participate in the regulation of ALA-induced anthocyanin accumulation (Fang et al. 2022; Zhang et al. 2024). Despite these advances, there remains much to be discovered about the mechanisms by which ALA promotes fruit coloration. For instance, the specific mechanisms through which MdMADS1 mediates ALA-induced anthocyanin accumulation are not yet fully understood. Unraveling these mechanisms is crucial for optimizing the use of ALA in fruit production and leads to significant advancements in our understanding of plant pigmentation.

In apple (*Malus* × *domestica*), the intricate mechanisms governing flavonoid biosynthesis have been extensively delineated. Key biosynthetic genes such as *MdCHS*, *MdCHI*, *MdDFR*, *MdANS*, and *MdUFGT*, along with the transporter gene *MdGSTF12*, are recognized for their pivotal roles in anthocyanin accumulation (Ju et al. 1999; Honda et al. 2002; Kim et al. 2003; Ubi et al. 2006; Ban et al. 2009; Fang et al. 2020). The regulatory impact of the well-characterized MYB-bHLH-WD40 (MBW) protein complexes on these gene expressions is particularly noteworthy (Sun et al. 2023). Moreover, an array of additional TFs has been implicated in the modulation of anthocyanin biosynthesis (Sun et al. 2021). For example, MdNAC52 (Sun et al. 2019), MdWRKY1 (Ma et al. 2021), MdWRKY11 (Liu et al. 2019b), MdWRKY40 (An et al. 2019; Zhang et al. 2019), MdWRKY41 (Mao et al. 2021), and MdWRKY71-like (Su et al. 2022) have been suggested to be crucial in apple anthocyanin biosynthesis. It appears that the coordinated regulation by many TFs, including WRKYs, leads to the production of anthocyanins in plants.

WRKYs, characterized by a conserved amino acid signature motif (WRKYGQK), are known to bind specifically to the W-box cis-element in the promoters of target genes (Chi et al. 2013). A wealth of research has established the involvement of WRKY TFs in a spectrum of plant processes, including growth, development, environmental adaptation, and disease resistance (Rushton et al. 2010; Chi et al. 2013). An expanding cohort of WRKY family members has been identified in the orchestration of plant anthocyanin accumulation, with examples such as PbWRKY75 in pear and StWRKY13 in potato, which enhance structural gene expression to promote biosynthesis (Cong et al. 2021; Zhang et al. 2022). This underscores the sophisticated control exerted by TFs over anthocyanin synthesis. In the context of apple, MdWRKY40 exemplifies this complexity, directly activating the transcription of *MdANS* and enhancing its binding affinity to target genes through interaction with MdMYB1, thereby upregulating anthocyanin biosynthesis in a multifaceted manner (Zhang et al. 2019). Conversely, MdWRKY41 is shown to inhibit anthocyanin synthesis by interacting with and reinforcing the repressive effect of MdMYB16 on key biosynthetic genes (Mao et al. 2021). These findings underscore the critical role of WRKY TFs in fruit anthocyanin biosynthesis, yet our understanding of their regulatory mechanisms remains in its nascent stages.

MADS-box TFs have also emerged as significant regulators of anthocyanin biosynthesis, with studies highlighting their influence on fruit coloration and ripening in various species (Fujisawa et al. 2011; Seymour et al. 2011; Wu et al. 2013). However, the precise role of MADS-box TFs in ALA-induced anthocyanin accumulation and its regulatory mechanisms remain largely unknown.

To dissect the regulatory mechanism of MdMADS1, a potential key player in ALA-induced anthocyanin accumulation in apple (Feng et al. 2016), we investigated its interactions with the promoters of structural genes and the known MBW components in apple anthocyanin biosynthesis (Espley et al. 2007; An et al. 2012; Xie et al. 2012; Zhang et al. 2023) in this study. Our findings indicate that while MdMADS1 activates the expression of genes like *MdCHS* and *MdUFGT*, it does not interact with MBW components, suggesting alternative regulatory pathways occur in ALA-induced anthocyanin biosynthesis. Employing a yeast two-hybrid screening approach with MdMADS1 as bait, we identified a WRKY TF MdWRKY71 as a novel interactor. Subsequent genetic and molecular interaction assays revealed that MdWRKY71 acts upstream of *MdMADS1*, activating its expression, and then they form different transcription factor complexes and cooperatively modulates the expression of downstream structural genes to enhance anthocyanin biosynthesis. Our discoveries introduce the MdWRKY71-MdMADS1 module as a refined regulatory mechanism distinct from the canonical MBW complex, providing novel insights into the sophisticated regulation of fruit anthocyanin biosynthesis. These findings not only expand our understanding of plant pigmentation but also hold promise for enhancing the breeding of high-quality fruit cultivars and improving fruit production practices.

## Results

### ALA induces *MdMADS1* expression during apple anthocyanin accumulation

**Fig. 1:**
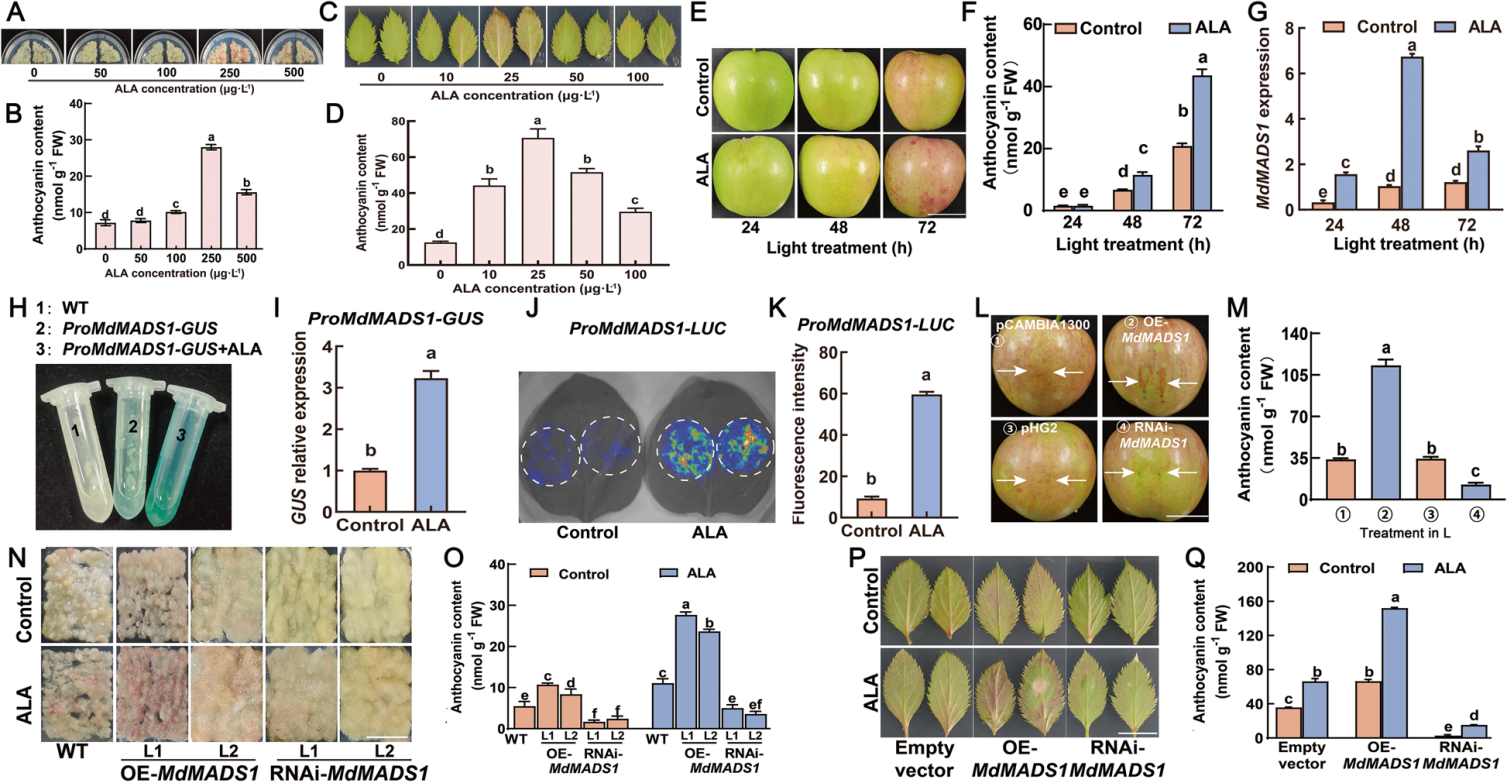
MdMADS1 is involved in regulation of ALA-induced anthocyanin synthesis. **A** and **B** Effect of ALA in different concentrations on anthocyanin accumulation in ‘Orin’ apple calli, which shows 250 μg L^−1^ is the optimal. **C** and **D** Effect of ALA in different concentrations on anthocyanin accumulation in ‘GL-3’ apple leaves, which shows 25 μg L^−1^ is the optimal. **E** ALA promotes anthocyanin accumulation in apple fruits. Debagged ‘Huashuo’ apples were treated with deionized water (Control) or 200 mg L^−1^ ALA in the dark. Subsequently, the fruits were continuously illuminated under 200 µmol m^−2^ s^−1^ light at 17 °C for 72 h. The representative pictures were taken. Scale bar, 4 cm. **F** The apple skin anthocyanin content in (**E**). **G** Relative expression of *MdMADS1* in apple fruit skin. *MdACTIN* was used as an internal reference. **H**-**I** GUS staining of ‘Orin’ calli transformed with the *ProMdMADS1-GUS,* which were treated with 250 μg L^−1^ ALA. *GUS* expression was detected by RT-qPCR. **J** The LUC assays in tobacco leaves show that 0.5 mg L^−1^ ALA activates the transcription of *MdMADS1*. **K** LUC fluorescence intensity in (**J**). **L** Apple skin injection test. The ‘Huashuo’ fruits were injected with *Agrobacterium* solutions carrying different vectors. Then, apple fruits were placed in an artificial climate chamber with constant light (200 µmol m^−2^ s^−1^) at 17 °C for 5 days. pCAMBIA1300: overexpression empty vector; OE-*MdMADS1*: overexpression of *MdMADS1*; pHG2: RNA interference empty vector; RNAi-*MdMADS1*: interference expression of *MdMADS1*. Scale bar, 4 cm. **M** The anthocyanin content of apple skin in (**L**). **N** Coloration phenotypes of ‘Orin’ calli of wild-type (WT), transgenic OE-*MdMADS1*, and RNAi-*MdMADS1* cultured on MS solid media containing 250 μg L^−1^ ALA or without ALA (Control) under 200 μmol m^−2^ s^−1^ constant light at 17 °C for half a month. L1 and L2 represent different apple transgenic callus cell lines. Scale bar = 1 cm. **O** The calli anthocyanin content in (**N**). **P** Coloration phenotypes of transient transgenic ‘GL-3’ apple leaves with 25 μg L^−^^1^ ALA or without (Control) for 3 days. Scale bar = 1 cm. **Q** The anthocyanin content in (**P**). The data are means ± SE of three biological replicates. The same small letters in each panel represent no significant differences *P* = 0.05

Feng et al. (2016) proposed that ‘Fuji’ apple calli can be used to study the effect of ALA on anthocyanin accumulation, which was verified by Zheng et al. (2021). Our result is also consistent with these reports (Fig. S1). However, we found that ‘Fuji’ calli were difficult to be sub-cultured, which is not conducive to stable genetic transformation. Therefore, we switched to ‘Orin’ calli instead. In a concentration effect test, we found that 250 μg L^−1^ ALA was the optimal for anthocyanin accumulation in ‘Orin’ calli (Fig. 1A, B). In another similar test, we observed 25 μg L^−1^ ALA was the optimal for ‘GL-3’ leaves to accumulate anthocyanins (Fig. 1C, D). It seems that anthocyanin accumulation in different tissues of apple needs different concentrations of ALA. In apple fruits, 200 mg L^−1^ ALA has been suggested to be suitable concentration for coloration (Xie et al. 2013; Fang et al. 2023). In current study, we used ALA at this dosage to treat the detached ‘Huashuo’ fruits, and found a significant enhancement of the anthocyanin content in the skin (Fig. 1E, F). These results substantiate that ALA can induce anthocyanin accumulation across apple tissues, including detached fruit skin, callus tissues derived from flesh, and detached leaves, although the suitable concentrations are quite different.

Feng et al. (2016) suggested a possible critical role of *MdMADS1* in ALA-induced anthocyanin accumulation by genetic manipulation of ‘Fuji’ apple. In the present study, we observed that ALA treatment induced an upregulation of *MdMADS1* expression in ‘Huashuo’ apple peel, which peaked significantly earlier than the anthocyanin accumulation (Fig. 1G). The temporal discrepancy suggests a potential correlation between ALA-induced anthocyanin accumulation and *MdMADS1* expression. To elucidate this relationship, we genetically transformed ‘Orin’ apple calli with the *ProMdMADS1-GUS* construct and subjected them to ALA treatment. Subsequent GUS staining revealed a substantial increase in GUS activity in the transgenic calli compared to the control (Fig. 1H, I). Furthermore, we constructed a reporter vector with the *MdMADS1* promoter driving the firefly luciferase gene (*LUC*) and performed transient transformations into tobacco leaves, with or without ALA treatment. Utilizing a live imaging system, we observed significantly enhanced fluorescence in ALA-treated leaves compared to the control (Fig. 1J, K). These observations suggest that the promoter of *MdMADS1* is responsive to ALA treatment, which can activate the gene expression.

Given the responsiveness of *MdMADS1* to ALA treatment, we explored its involvement in anthocyanin biosynthesis within the apple peel. Transient overexpression (OE-*MdMADS1*) and RNA interfering (RNAi-*MdMADS1*) of *MdMADS1* in apple skin were conducted. The results showed that OE-*MdMADS1* promoted fruit coloration and anthocyanin accumulation, while RNAi-*MdMADS1* had an opposite effect (Fig. 1L, M). Stable transformation of ‘Orin’ calli with OE-*MdMADS1* or RNAi-*MdMADS1* constructs, which were confirmed by PCR and RT-qPCR (Figure S2), mirrored the transient transformations, with overexpression enhancing and RNAi inhibiting anthocyanin accumulation in callus tissues (Fig. 1N, O). It was noticed that ALA treatment uniformly promoted anthocyanin accumulation across all genotypes, including the wild type, OE, and RNAi lines of *MdMADS1*. Consistent with these, when these constructs were transiently transformed into apple leaves, we got the same results (Fig. 1P, Q). These comprehensive findings confirm that MdMADS1 is an indispensable and positive regulatory factor in the ALA-promoted anthocyanin biosynthesis pathway in apple. Since ALA can partially relieve the inhibition of RNAi-*MdMADS1*, there must be other factors involved in the ALA-induced anthocyanin accumulation.

### MdMADS1 activates the expression of two anthocyanin biosynthetic genes *MdCHS *and *MdUFGT*

**Fig. 2:**
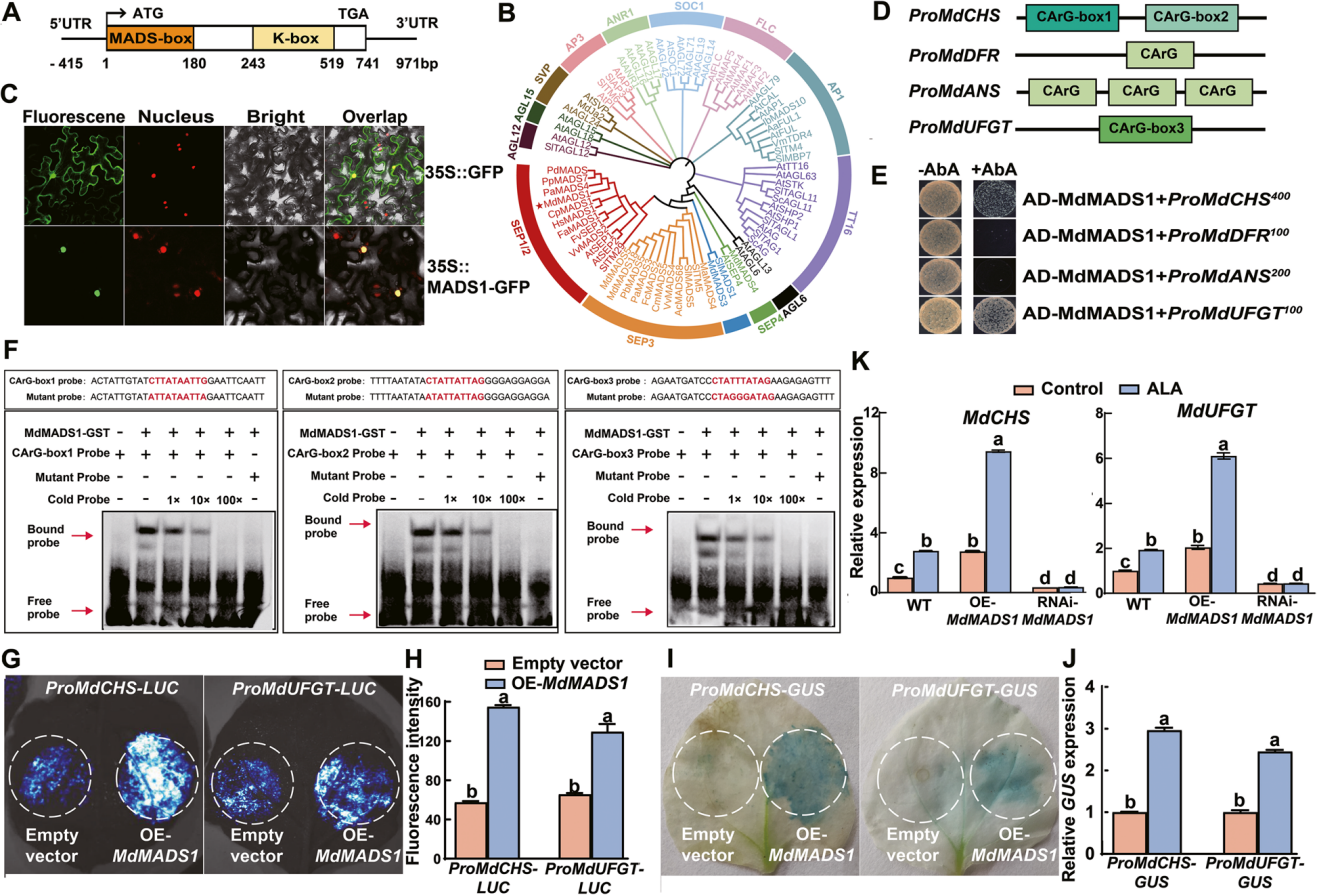
MdMADS1 activates the transcription of *MdCHS* and *MdUFGT*. **A** The coding sequence (CDS) length of *MdMADS1* is 741 bp and the 5' UTR and the 3' UTR are indicated by the black lines. The orange and yellow rectangles represent the MADS-box and K-box domains, respectively. The numbers in the figure are the positions relative to the *MdMADS1* from the start codon ATG. **B** Phylogenetic tree analysis using amino acid sequences of MdMADS1 and other species MADS-box members. All species are as follows. At: Arabidopsis (*Arabidopsis thaliana*), Sl: tomato (*Solanum lycopersicum*), Pp: peach (*Prunus persica*), Md: apple (*Malus* x *domestica*), Vv: grape (*Vitis vinifera*), Ib: sweet potato (*Ipomoea batatas*), Aa: anthurium (*Anthurium andraeanum*), Ac: kiwifruit (*Actinidia chinensis*), Vm: bilberry (*Vaccinium myrtillus*)*,* Sc: cineraria (*Senecio cruentus*), Ma: banana (*Musa acuminata*), Cm: melon (*Cucumis melo*), Fc: strawberry (*Fragaria chiloensis*), Pa: cherry (*Prunus avium*), Pb: pear (*Pyrus bretschneideri*), Fa: strawberry (*Fragaria* x *ananassa*), Fv: woodland strawberry (*Fragaria vesca*), Hs: roselle (*Hibiscus sabdariffa*), Cp: papaya (*Carica papaya*), Pd: almond (*Prunus dulcis*). **C** Subcellular localization of MdMADS1. 35S::GFP and 35S::MdMADS1-GFP were transiently expressed in tobacco leaves. The GFP fluorescence was observed via confocal microscopy. **D** Schematic diagram showing MADS-box binding cis-elements (CArG-boxes) in the promoters of *MdCHS*, *MdDFR*, *MdANS*, and *MdUFGT*. **E** Y1H experiment analyzes the interactions between MdMADS1 and promoters of *MdCHS*, *MdDFR*, *MdANS*, and *MdUFGT*. The Aureobasidin A (AbA) concentrations are the number in superscript. **F** EMSA shows that MdMADS1-GST fusion protein binds directly to the CArG-box1 and CArG-box2 in the *MdCHS* promoter, and CArG-box3 in the *MdUFGT* promoter. The cold probe was a non-labeled competitive probe. –, absence of relevant probes or proteins; + , presence of relevant probes or proteins. 1 × , 10 × , and 100 × represent the amount of competitive probe added, which is onefold, tenfold, and 100-fold of the binding probe, respectively. **G** The luciferase reporter assays show that MdMADS1 activates the transcription of *MdCHS* and *MdUFGT*. **H** LUC fluorescence intensity corresponding to the assays in (**G**) **I** GUS staining of tobacco leaves reveals that MdMADS1 activates the transcription of *MdCHS* and *MdUFGT*. **J** The relative expression of *GUS* determined by RT-qPCR. **K** The relative expression of *MdCHS* and *MdUFGT* in WT and transgenic apple calli treated with ALA. The data are means ± SE of three biological replicates. Different lowercase letters in each panel represent significant differences at *P* = 0.05

To elucidate the function of MdMADS1, we initiated our study with a comprehensive analysis of its gene structure and amino acid sequence. It was found that the coding sequence (CDS) of *MdMADS1* spans 741 bp, encoding 246 amino acids (Fig. 2A). The amino acid sequence of MdMADS1 contains the highly conserved MADS-box, the K-box, C-region, and I-region, exhibiting high homology with a multitude of MADS-box transcription factors, such as CpMADS1 of papaya (*Carica papaya*), VvMADS2 of grape (*Vitis vinifera*), AtSEP1, and AtSEP2 of Arabidopsis (Figure S3). Phylogenetic analysis positioned MdMADS1 within the SEP1/2 subgroup (Fig. 2B), with the closest genetic relationship with PaMADS4 of cherry (*Prunus avium*), PpMADS7 of peach (*Prunus persica*) and PdMADS of almond (*Prunus dulcis*). Subcellular localization assays, utilizing the recombinant MdMADS1-GFP vector in tobacco leaves, confirmed the nuclear localization of MdMADS1 (Fig. 2C), thereby establishing its identity as a canonical MADS-box transcription factor.

In our quest to unravel the regulatory influence of MdMADS1 on anthocyanin biosynthesis genes, we identified at least one CArG-box in the promoters of key biosynthetic genes, including *MdCHS*, *MdDFR*, *MdANS*, and *MdUFGT*, implying a potential direct regulatory mechanism (Fig. 2D). However, when we conducted yeast one-hybrid (Y1H) assay, co-transforming *pGADT7-MdMADS1* with various anthocyanin biosynthetic gene promoters fused to the pAbAi vector, the results showed the binding affinity of MdMADS1 for the promoters of *MdCHS* and *MdUFGT* but not *MdANS* and *MdDFR* (Fig. 2E). Additionally, we assessed the binding capacity of MdMADS1 to the promoters of additional anthocyanin biosynthetic and transporter genes, including *MdCHI*, *MdF3H*, *MdDFR*, *MdANS*, and *MdGSTF12*, but no binding was found between them (Figure S4). These suggest that MdMADS1 specifically binds to the promoters of *MdCHS* and *MdUFGT*.

To pinpoint the binding sites within these promoters, we employed EMSA with biotin-labeled probes. Both *ProMdCHS* and *ProMdUFGT*, known to harbor CArG-boxes, were subjected to this analysis. The EMSA demonstrated that MdMADS1 bound to the labeled probes, with binding affinity diminishing in the presence of increasing concentrations of cold probes for both promoters. Notably, the absence of a binding band with mutant probes validated the specificity of the interaction at the CArG-boxes (Fig. 2F).

Subsequently, we used a transient transactivation assay in tobacco leaves to evaluate the impact of MdMADS1 on the transcriptional regulation of *MdCHS* and *MdUFGT*. We constructed the effector plasmid *35S::MdMADS1* and the reporters *ProMdCHS-LUC* and *ProMdUFGT-LUC*, respectively. As expected, LUC assays revealed that MdMADS1 significantly enhanced the activity of both promoters by 150% and 110%, respectively, over the control with the empty vector (Fig. 2G, H). To corroborate these findings, we performed *GUS* reporter gene assays with the corresponding *ProMdCHS-GUS* and *ProMdUFGT-GUS* constructs. In alignment with the LUC assay outcomes, MdMADS1 was found to activate the promoters of both *MdCHS* and *MdUFGT* (Fig. 2I, J). In addition, we measured the relative expression levels of *MdCHS* and *MdUFGT* in transgenic ‘Orin’ calli. The results showed that the expression levels of *MdCHS* and *MdUFGT* were significantly increased in OE-*MdMADS1* calli, while significantly decreased in RNAi-*MdMADS1* calli (Fig. 2K). Collectively, these results underscore the role of MdMADS1 in accelerating anthocyanin biosynthesis through the direct activation of key structural genes, *MdCHS* and *MdUFGT*.

### MdMADS1 interacts with a WRKY transcription factor MdWRKY71

Anthocyanin biosynthesis is orchestrated by a complex interplay of multiple TFs, rather than the action of a single regulator (Espley et al. 2007; Li et al. 2020; Mao et al. 2021). To screen the TFs interacting with MdMADS1, we initially explored potential interactions with components of the MYB, bHLH, and WD40 families, which are known for forming the classic MBW complex. Utilizing yeast two-hybrid (Y2H) and bimolecular fluorescence complementation (BiFC) assays, we found no interactions between MdMADS1 and the reported MBW complex members, including MdMYB10, MdbHLH3, MdbHLH33, and MdTTG1 (Figure S5). This suggests that the regulatory role of MdMADS1 in apple anthocyanin accumulation operates independently of the MBW complex.

**Fig. 3:**
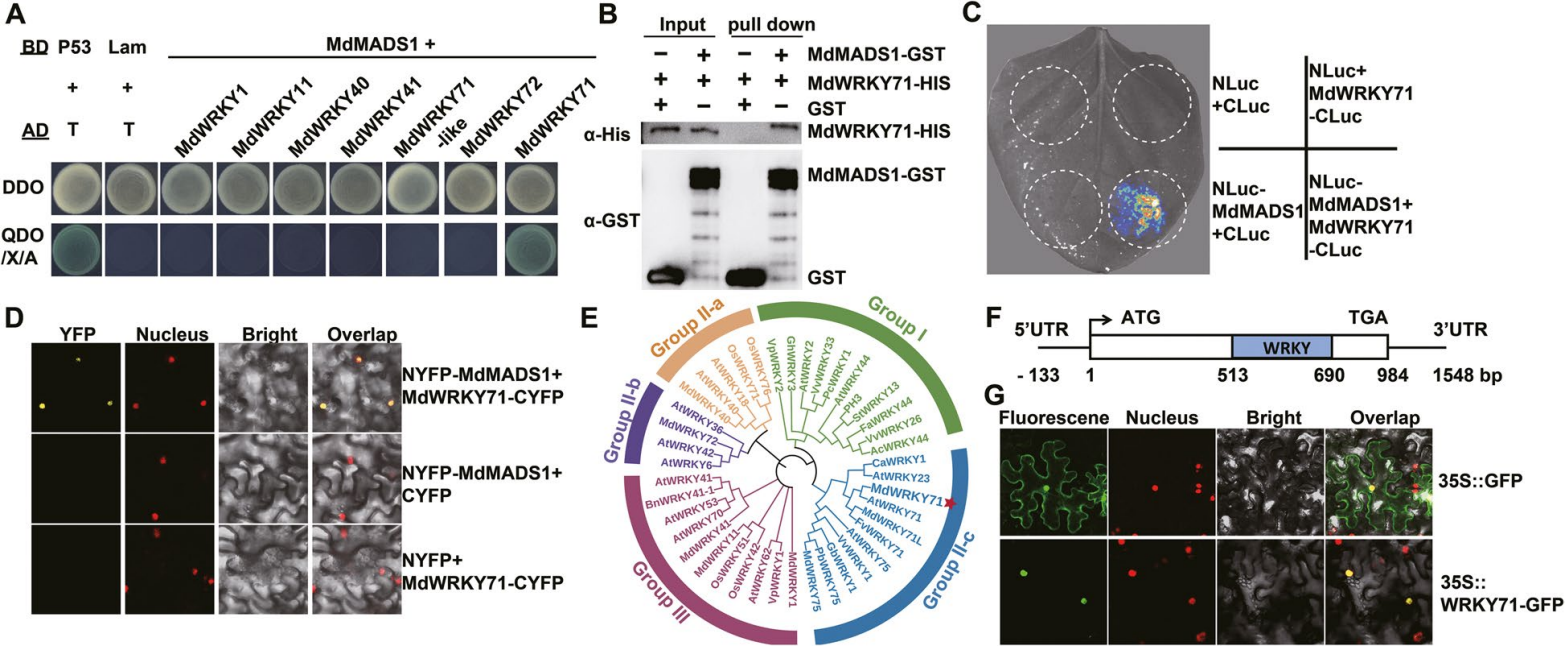
Protein interaction between MdMADS1 and MdWRKY71. **A** Y2H assays show that MdMADS1 interacts with the MdWRKY71 protein in yeasts. DDO: SD/ − Trp/ − Leu medium; QDO/X/A: SD/ − Trp/ − Leu/ − Ade/ − His medium with X-α-gal and Aureobasidin A (AbA). **B** Pull-down experiments show that MdMADS1 interacts with MdWRKY71. GST or MdMADS1-GST proteins expressed in *E. coli* were incubated with resin containing immobilized His-tagged MdWRKY71 protein. Western blot analysis was conducted using antibodies against the His-tag and GST-tag. **C** LCI assay confirms the interaction of MdMADS1 with MdWRKY71. **D** BiFC assay verifies the interaction of MdMADS1 with MdWRKY71 in plant cells. Confocal microscopy was used to view the fluorescence. The red fluorescence is a marker for nuclear localization. **E** Phylogenetic relationship of MdWRKY71 with selected WRKY proteins from other species. All species are as follows. At: Arabidopsis (*Arabidopsis thaliana*), Sl: tomato (*Solanum lycopersicum*), Pb: pear (*Pyrus bretschneideri*), Vv: grape (*Vitis vinifera*), Md: apple (*Malus* x *domestica*), Ib: sweet potato (*Ipomoea batatas*), Fa: strawberry (*Fragaria* x *ananassa*), Bn: canola (*Brassica napus*), Gb: cotton (*Gossypium barbadense*), Os: rice (*Oryza sativa*), Vp: East China grape (*Vitis pseudoreticulata*), Fv: woodland strawberry (*Fragaria vesca*), Ca: pepper (*Capsicum annuum*), Gh: cotton (*Gossypium hirsutum*), Pc: parsley (*Petroselinum crispum*). **F** The coding sequence (CDS) of *MdWRKY71* is 984 bp. Black lines indicate the 5' UTR and the 3' UTR, respectively. The WRKY domain is depicted by the blue rectangle. The numbers indicate the positions relative to the start codon ATG of *MdWRKY71*. **G** Subcellular localization of the MdWRKY71 protein. 35S::GFP and 35S::MdWRKY71-GFP were transformed into tobacco leaves. The GFP fluorescence was observed using confocal microscopy

Subsequently, we conducted a Y2H screening with the full-length MdMADS1 protein as bait to identify other factors that might participate in ALA-promoted anthocyanin biosynthesis. This screen yielded 12 candidate proteins capable of interacting with MdMADS1 (Table S2). Notably, a cDNA fragment (MD10G1266400/XM_008385286.3) encoding a WRKY domain protein, designated as MdWRKY71, was identified. The Y2H assay confirmed the interaction between the MdMADS1 and MdWRKY71 proteins (Fig. 3A), but other MdWRKY TFs including MdWRKY1, MdWRKY11, MdWRKY40, MdWRKY41, MdWRKY71L, and MdWRKY72, which have been reported involved in the regulation of anthocyanin biosynthesis, did not interacted with MdMADS1. The results highlight a specific interaction between MdMADS1 and MdWRKY71.

In order to verify the interaction, we performed in vitro pull-down experiments with MdMADS1-GST and MdWRKY71-HIS proteins expressed and purified from *E. coli* BL21. The subsequent electrophoresis results demonstrated that GST-tagged MdMADS1 interacted with HIS-tagged MdWRKY71 (Fig. 3B). Furthermore, the MdMADS1-MdWRKY71 interaction was corroborated by the luciferase complementation imaging (LCI) assay (Fig. 3C) and the BiFC assay (Fig. 3D) in tobacco leaves, with a strong yellow fluorescent signal observed exclusively in the nucleus of co-transformed cells. Together, these findings show that MdWRKY71 directly interacts with MdMADS1.

To elucidate the evolutionary relationship of MdWRKY71 with its orthologs, a phylogenetic tree was constructed using the neighbor-joining (NJ) method. The analysis reveals that MdWRKY71 is most closely related to AtWRKY71, FvWRKY71, and MdWRKY71L within Group II-c (Fig. 3E). To investigate the function of MdWRKY71, its CDS was amplified from ‘Fuji’ apple peels, which is 984 nucleotides in length and encode a 327 amino acid protein (Fig. 3F). The deduced protein sequence features a characteristic WRKY domain (WRKYGQK) and a C-terminal zinc finger motif (Figure S6). The subcellular localization of the MdWRKY71 protein was determined by transient expression of the recombinant MdWRKY71-GFP fusion protein in tobacco leaves, confirming its nuclear localization (Fig. 3G). These findings collectively indicate that MdWRKY71 is a bona fide WRKY TF.

### MdWRKY71 contributes to ALA-promoted anthocyanin biosynthesis

**Fig. 4:**
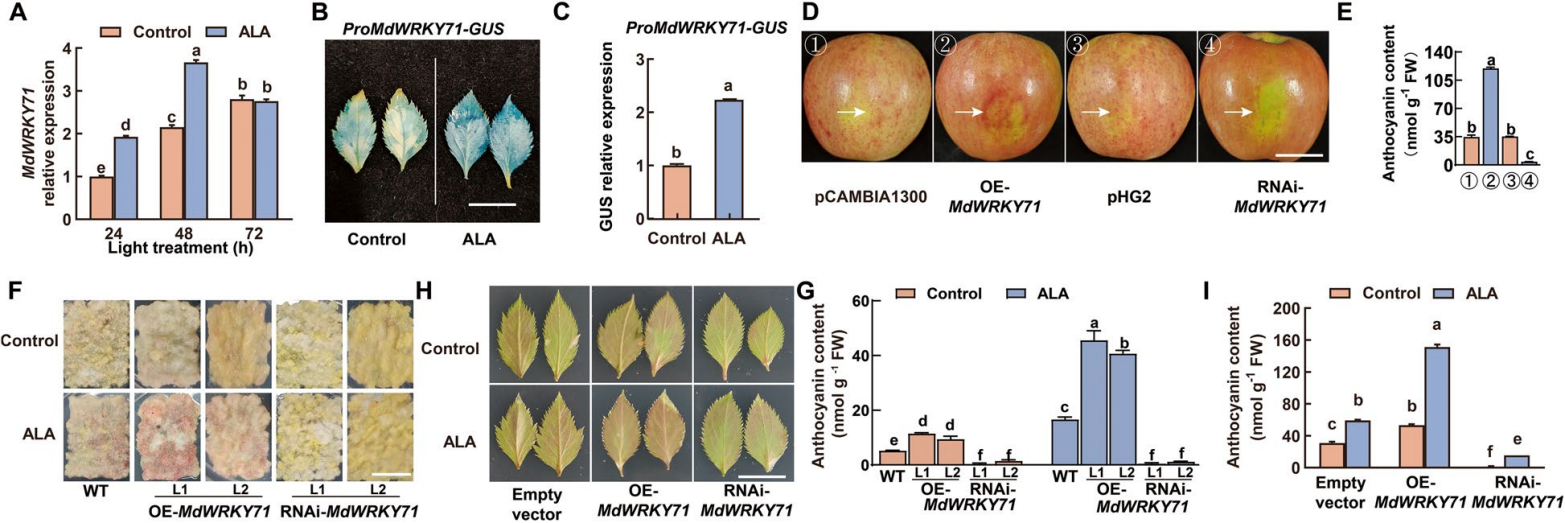
MdWRKY71 promotes ALA-induced anthocyanin accumulation. **A** The expression levels of *MdWRKY71* in ‘Huashuo’ apple skin treated with 200 mg L^−1^ ALA at different time points. **B** and **C** GUS staining of ‘GL-3’ leaves transiently transformed with *ProMdWRKY71-GUS*, cultured in the dark on MS solid media containing 25 μg L^−1^ ALA or without (Control) for 24 h, then transferred to a chamber with constant light (200 µmol m^−2^ s^−1^) for 72 h. *GUS* expression was analyzed by RT-qPCR analysis. Scale bar = 1 cm. **D** Apple skin injection test. ‘Huashuo’ fruits were harvested at green mature stage, and injected with different vector *Agrobacterium* solutions, kept in the dark for 12 h. Then, they were transferred to a chamber with constant light (200 µmol m^−2^ s^−1^) at 17 °C for 5 days. pCAMBIA1300: overexpression empty vector; OE-*MdWRKY71*: overexpression of *MdWRKY71*; pHG2: RNA interference empty vector; RNAi-*MdWRKY71*: interference expression of *MdWRKY71*. Scale bar = 4 cm. **E** The apple skin anthocyanin content in (**D**). **F** Coloration phenotype of two-week-old apple ‘Orin’ calli of WT, transgenic OE-*MdWRKY71*, and RNAi-*MdWRKY71* treated with 250 μg L^−1^ ALA or without (Control) at 17 °C under 200 µmol m^−2^ s^−1^ light for half a month. L1 and L2 represent different lines of apple transgenic callus cells. Scale bar = 1 cm. **G** The callus anthocyanin content in (**F**). **H** Coloration phenotypes of apple leaves treated with ALA or without (Control). ‘GL-3’ apple leaves were plated on medium supplemented with 25 μg L^−1^ ALA and then cultured under a constant light of 200 µmol m^−2^ s^−1^ chamber at 17 °C. Scale bar = 1 cm. **I** The leaf anthocyanin content in (**H**). Data are means ± SE of three biological replicates. Different lowercase letters in each panel indicate significant differences *P* = 0.05

To ascertain the role of *MdWRKY71* in ALA-induced anthocyanin biosynthesis, we examined its expression patterns in apple fruits following ALA treatment in Fig. 1E. It was found that the transcription of *MdWRKY71* exhibited a notable up-regulation 24 h after ALA treatment, reaching a peak at 48 h (Fig. 4A). Furthermore, the introduction of the *MdWRKY71* promoter construct, *ProMdWRKY71-GUS*, into wild-type apple leaves, followed by ALA treatment, led to a substantial enhancement in GUS activity as revealed by histochemical staining and *GUS* expression levels (Fig. 4B, C). These results demonstrate that *MdWRKY71* expression is responsive to ALA treatment and MdWRKY71 may play an important role in ALA-induced anthocyanins accumulation.

In order to validate the function of MdWRKY71, we constructed overexpression (OE-*MdWRKY71*) and RNA interference (RNAi-*MdWRKY71*) expression vectors. Transient transformation of apple skin with these constructs revealed that OE-*MdWRKY71* significantly enhanced anthocyanin accumulation, whereas RNAi-*MdWRKY71* substantially reduced it (Fig. 4D, E). To further confirm the role of MdWRKY71 in the context of ALA treatment, we stably integrated OE-*MdWRKY71* and RNAi-*MdWRKY71* into apple calli (Figure S7) and then subjected the transgenic calli to ALA treatment. The results demonstrated that OE-*MdWRKY71* increased, while RNAi-*MdWRKY71* decreased the anthocyanin content in apple calli (Fig. 4F, G), underscoring the positive regulatory function of MdWRKY71 in anthocyanin biosynthesis. Additionally, exogenous ALA upregulated anthocyanin accumulation in the WT and OE-*MdWRKY71*, but not RNAi-*MdWRKY71*, suggesting that ALA-induced anthocyanin accumulation might be dependent on *MdWRKY71* expression. However, when we transiently transformed the OE-*MdWRKY71* or RNAi-*MdWRKY71* vector into apple leaves and treated with ALA, the results were similar with the calli transformation assay, but ALA can promote anthocyanin accumulation in the RNAi-*MdWRKY71* (Fig. 4H, I). Collectively, these results establish MdWRKY71 as a pivotal, but not only, positive regulator in the ALA-induced anthocyanin biosynthetic pathway in apple.

### MdWRKY71 potentiates the transcriptional activation of MdMADS1 on downstream genes

**Fig. 5:**
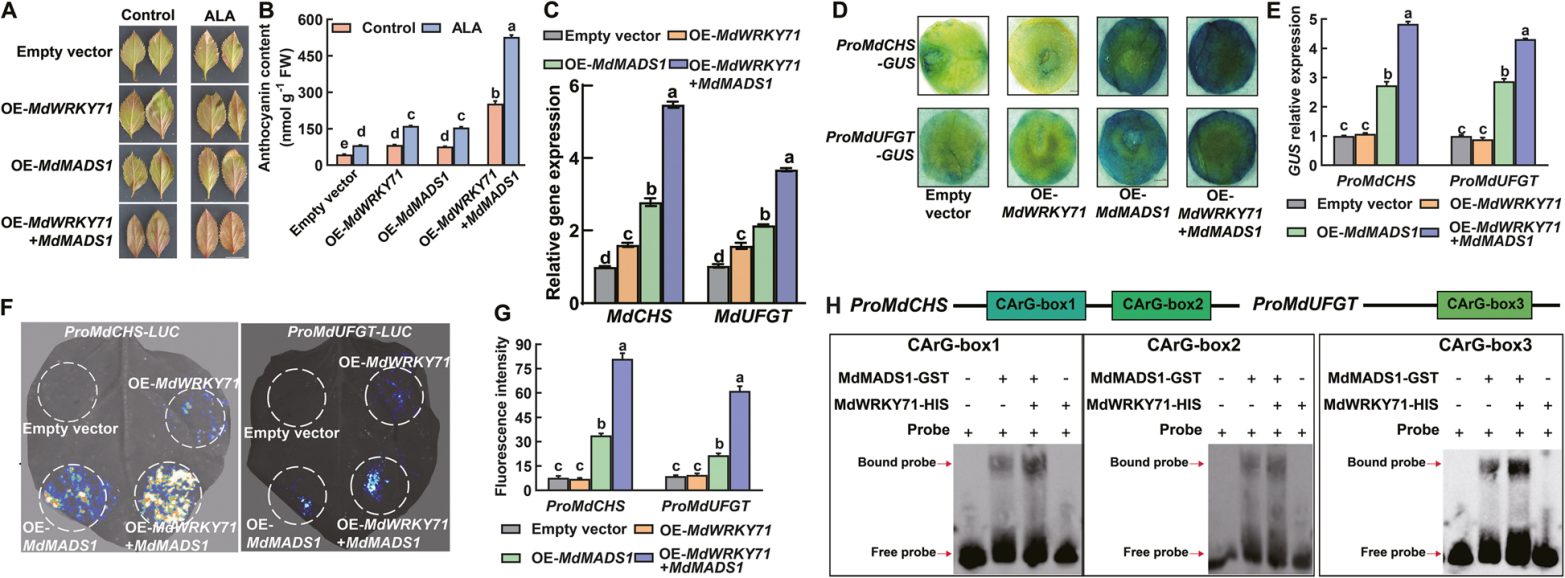
MdWRKY71 promotes the transcriptional activity of MdMADS1 on *MdCHS* and *MdUFGT*. **A** Coloration phenotypes of transient transgenic ‘GL-3’ apple leaves treated with 25 μg L^−^^1^ ALA or without (Control) for 3 days. Scale bar = 1 cm. **B** Comparison of the leaf anthocyanin content in (**A**). OE-*MdWRKY71*: *MdWRKY71*-overexpression line; OE-*MdMADS1*: *MdMADS1*-overexpression line; OE-*MdMADS1* + OE-*MdWRKY71*: transgenic lines overexpressing both *MdMADS1* and *MdWRKY71*. **C** The relative expression of *MdCHS* and *MdUFGT* in transgenic apple leaves. **D** GUS staining of tobacco leaves reveals that MdWRKY71 enhances the regulatory activity of MdMADS1 to activate the expression of *MdCHS* and *MdUFGT*. **E ***GUS* expression in (**D**) was detected by RT-qPCR. **F** The luciferase reporter assays in tobacco leaves show that MdWRKY71 enhances the regulatory activity of MdMADS1 to activate the expression of *MdCHS* and *MdUFGT*. **G** LUC fluorescence intensity in (**F**). **H** Effect of MdWRKY71 on the binding strength of MdMADS1 to *CArG-box* elements on *MdCHS* and *MdUFGT* promoters using EMSA. Data are means ± SE of three biological replicates. Different lowercase letters in each panel represent significant differences *P* = 0.05

Given the aforementioned findings, we hypothesized that the interaction between MdMADS1 and MdWRKY71 might potentiate their regulatory roles in anthocyanin biosynthesis. To explore this possibility, we conducted transient co-expression assays of both genes in apple leaves. Our results revealed that the leaves co-expressing OE-*MdWRKY71* and OE*-MdMADS1* exhibited a higher anthocyanin content compared to those transformed with either OE-*MdWRKY71* or OE-*MdMADS1* alone (Fig. 5A, B). Furthermore, exogenous ALA led to a pronounced increase in anthocyanin levels, indicating a synergistic effect between the two transcription factors on anthocyanin biosynthesis, which is further amplified by ALA. In addition, we measured the expression levels of *MdCHS* and *MdUFGT* in the transiently transformed leaves. The results showed that compared with the single transformation of OE-*MdMADS1* or OE-*MdWRKY71*, co-expression of OE-*MdWRKY71* and OE-*MdMADS1* significantly increased the expression of *MdCHS* and *MdUFGT* (Fig. 5C). These results indicate that MdWRKY71 has a synergistic effect with MdMADS1 on the downstream target gene transcription.

Subsequently, we investigated the impact of MdWRKY71 on the binding of MdMADS1 to the promoters of *MdCHS* and *MdUFGT* using *GUS* and *LUC* reporter gene assays. The tobacco leaves overexpressing *MdWRKY71* (OE-*MdWRKY71*) alone did not show a difference in color from the control, implying that MdWRKY71 cannot directly activate these promoters (Fig. 5D, E). In contrast, the leaves overexpressing *MdMADS1* (OE-*MdMADS1*) displayed a blue color, indicative of its ability to activate the promoters of *MdCHS* and *MdUFGT*. Strikingly, co-transformation with OE-*MdWRKY71* and OE-*MdMADS1* resulted in a significantly darker blue color, suggesting that MdWRKY71 enhances the binding of MdMADS1 to these promoters, thereby upregulating gene expression. This synergistic effect was corroborated by the LUC assay in tobacco leaves (Fig. 5F, G). Additionally, EMSA experiments demonstrated that the presence of the MdWRKY71 protein significantly heightened the binding affinity of MdMADS1 to its target genes (Fig. 5H). In summary, these findings suggest that the interaction between MdMADS1 and MdWRKY71 promotes the binding of MdMADS1 to the promoters of downstream genes *MdCHS* and *MdUFGT*, thereby facilitating the gene expression and anthocyanin biosynthesis.

### MdWRKY71 directly activates *MdMADS1* expression

**Fig. 6:**
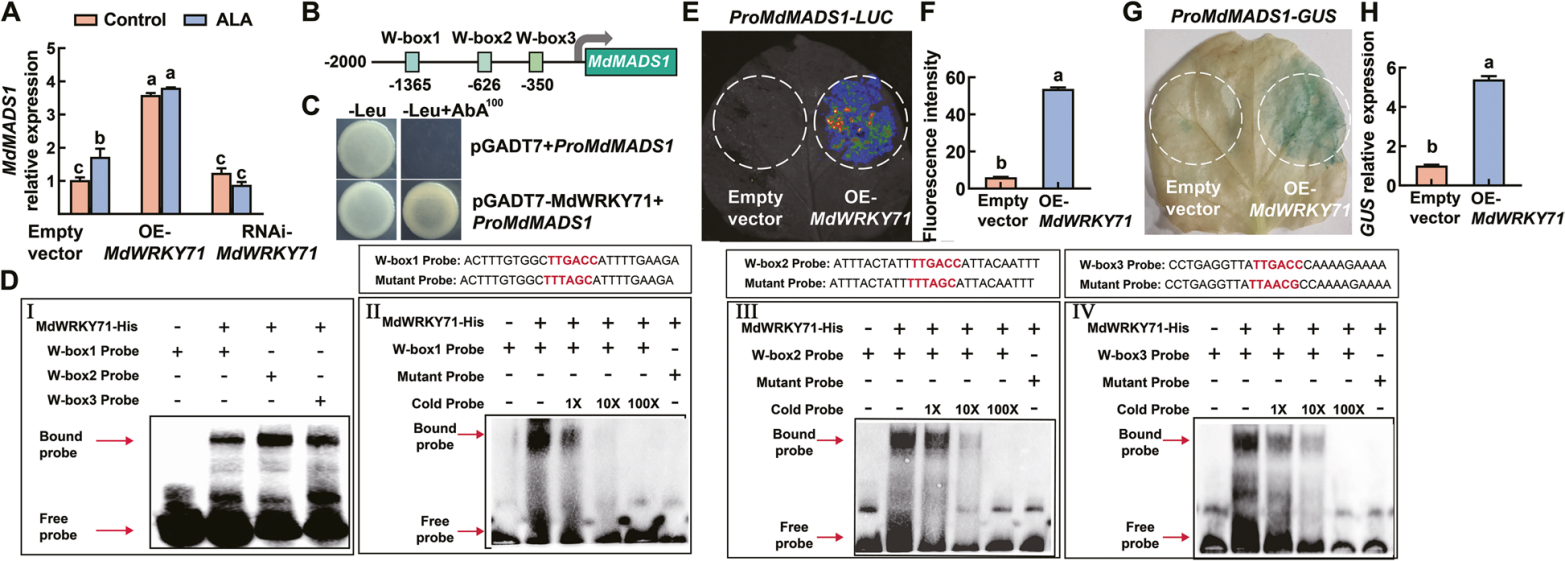
MdWRKY71 activates *MdMADS1* expression. **A** Transcriptional levels of *MdMADS1* in transient transgenic ‘GL-3’ apple leaves. Empty vector, OE-*MdWRKY71*, and RNAi-*MdWRKY71* transiently transgenic leaves were treated by 25 μg L^−1^ ALA and then cultured in a constant light of 200 µmol m^−2^ s^−1^ chamber at 17 °C for 3 d. **B** Diagram of the *MdMADS1* promoter. Three W-boxes are marked with pale green boxes. **C** Y1H assay shows interaction of MdWRKY71 with *MdMADS1* promoter. **D** EMSA shows MdWRKY71-His binds to the W-box elements of the *MdMADS1* promoter, where I show that MdWRKY71 can bind to W-Box1, W-box2, and W-box3, respectively, while II, III, and IV show MdWRKY71 can specifically and stoichiometrically bind to W-Box1, W-box2, and W-box3, respectively. The W-box probe was a biotin-labeled fragment containing the W-box element. –, absence of relevant probes or proteins; + , presence of relevant probes or proteins. 1 × , 10 × , and 100 × represent the amount of competitive probe added, which is onefold, tenfold, and 100-fold of the binding probe, respectively. **E** The fluorescence intensity in tobacco leaves indicates that MdWRKY71 activates the *MdMADS1* promoter. **F** LUC fluorescence intensity level in (**E**). **G** GUS staining of tobacco leaves reveals that MdWRKY71 enhances the transcription activity of the *MdMADS1* promoter. **H** The relative expression of *GUS* in (**G**) detected by RT-qPCR. Data are means ± SE of three biological replicates. Different lowercase letters in each panel represent significant differences *P* = 0.05

Considering the characteristics of the WRKY TF, we were intrigued by the possibility that MdWRKY71 might exert regulatory control over the expression of *MdMADS1*. To explore this potential regulatory link, we assessed the expression level of *MdMADS1* in apple leaves that had been transiently transformed with constructs overexpressing (OE-) or RNA interfering (RNAi-) *MdWRKY71*. Our results indicated a significant upregulation of *MdMADS1* with elevated MdWRKY71 levels and a corresponding downregulation with reduced MdWRKY71 levels, however, ALA did not affect the effect of MdWRKY71 on *MdMADS1* expression (Fig. 6A). These findings imply that MdWRKY71 plays a dominant role in modulating *MdMADS1* expression.

Subsequently, we scrutinized the 2000 bp promoter region of *MdMADS1* and identified three W-box elements (Fig. 6B), which are known as potential binding sites for WRKY transcription factors. To substantiate the interaction between MdWRKY71 and the *MdMADS1* promoter, a series of assays were conducted, including Y1H, EMSA, LUC, and GUS reporter. The Y1H assay confirmed the binding of MdWRKY71 to the promoter (Fig. 6C). Next, using fragments containing three W-box elements on the *MdMADS1* promoter as DNA probes, we performed EMSA experiments. We found that MdWRKY71 can bind to these three W-box probes (Fig. 6D-I), and the interaction gradually weakened with the addition of competitive probes. When the probes were muted, no binding of MdWRKY71 was detected (Fig. 6D-II, III, and IV). These suggest that the W-box elements are essential for MdWRKY71 binding to *MdMADS1* promoter. The LUC and GUS reporter assays further showed that MdWRKY71 significantly promoted the transcription activity of *MdMADS1* promoter (Fig. 6E-H), confirming its ability to directly bind the promoter and activate the expression of *MdMADS1*. In contrast, we found no binding of MdMADS1 to the promoter of *MdWRKY71* in Y1H and GUS assays (Figure S8), indicating that the regulatory influence is unidirectional, with MdWRKY71 positioned upstream in the regulatory cascade, acting as a TF to modulate *MdMADS1* expression.

### MdWRKY71 directly binds to the promoters of *MdANS *and *MdDFR* to promote anthocyanin biosynthesis

**Fig. 7:**
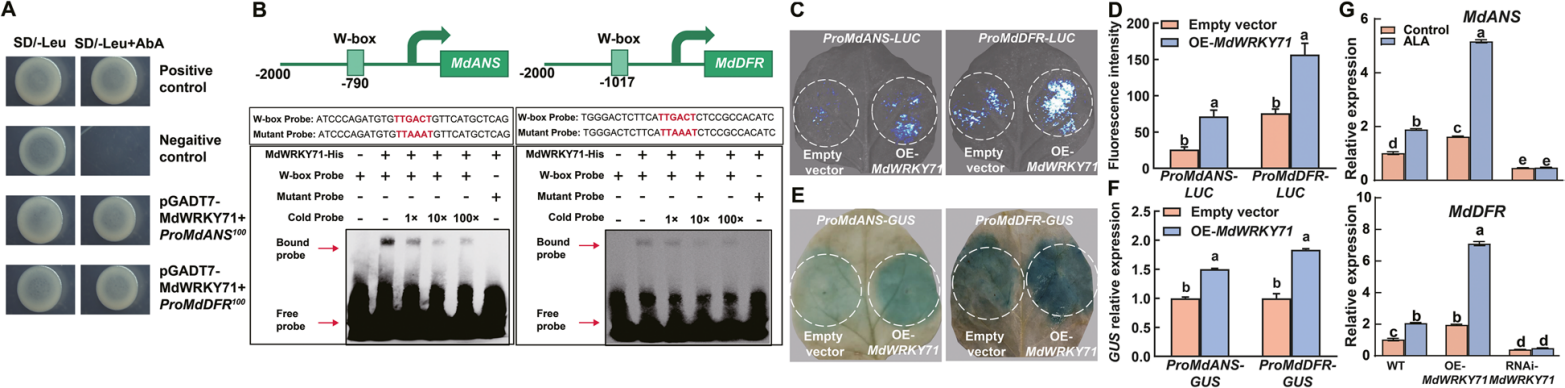
MdWRKY71 binds to the promoters of *MdANS* and *MdDFR* to activate the downstream gene expression. **A** Y1H assays show the interaction of MdWRKY71 with the *MdANS* and *MdDFR* promoters. The superscript numbers in the upper right corner represent AbA concentrations. **B** Diagram of the *MdANS* and *MdDFR* promoters and EMSA. The W-box elements are marked with pale green boxes. The EMSA shows MdWRKY71-His binds to the W-box elements of the *MdANS* and *MdDFR* promoters. The cold probe was a non-labeled competitive probe. –, absence of relevant probes or proteins; + , presence of relevant probes or proteins. 1 × , 10 × , and 100 × represent the amount of competitive probe added, which is onefold, tenfold, and 100-fold of the binding probe, respectively. **C** The luciferase reporter assays in tobacco leaves show that MdWRKY71 activates the *MdANS* and *MdDFR* promoters. **D** LUC fluorescence intensity level in (**C**). **E** GUS staining of tobacco leaves reveals that MdWRKY71 activates the promoters of *MdANS* and *MdDFR*. **F ***GUS* expression in (**E**) was analyzed by RT-qPCR analysis, and the value of EV was set to 1 as a reference control. **G** The relative expression levels of *MdANS* and *MdDFR* in WT and transgenic apple calli treated with ALA. Data are means ± SE of three biological replicates. Different lowercase letters in each panel represent significant differences *P* = 0.05

In addition to its established role in regulating the expression of *MdMADS1* and augmenting transcriptional activity through interaction with this MADS-box transcription factor, the capacity of MdWRKY71 to directly modulate anthocyanin biosynthesis by binding to the promoters of key biosynthetic genes is an open question. To address this, we utilized the Y1H system to assess the binding affinity of MdWRKY71 for a suite of anthocyanin pathway genes. Y1H results indicated that MdWRKY71 specifically interacts with the promoters of *MdANS* and *MdDFR* (Fig. 7A), while exhibiting no binding to other genes in the pathway, such as *MdCHS*, *MdCHI*, *MdF3H*, *MdUFGT* and *MdGSTF12* (Figure S9).

Subsequent EMSA analyses provided confirmatory evidence of the direct binding of MdWRKY71 to the W-box element (TTGACT) within the promoters of both *MdANS* and *MdDFR* (Fig. 7B). The *LUC* and *GUS* reporter gene assays further demonstrated a significant enhancement in the promoter activities of *MdANS* and *MdDFR* in the presence of MdWRKY71 (Fig. 7C-F). In addition, overexpression of *MdWRKY71* increased the expression of *MdANS* and *MdDFR*, while interference with *MdWRKY71* showed an opposite effect (Fig. 7G). These integrated findings collectively suggest that MdWRKY71 possesses the intrinsic ability to bind directly to the promoters of anthocyanin biosynthetic genes *MdANS* and *MdDFR*, thereby exerting a positive regulatory effect on anthocyanin biosynthesis. This direct regulatory mechanism expands our understanding of the complex transcriptional network governing pigment production in apples.

### Interaction of MdWRKY71 with MdMADS1 promotes binding to the downstream genes

**Fig. 8:**
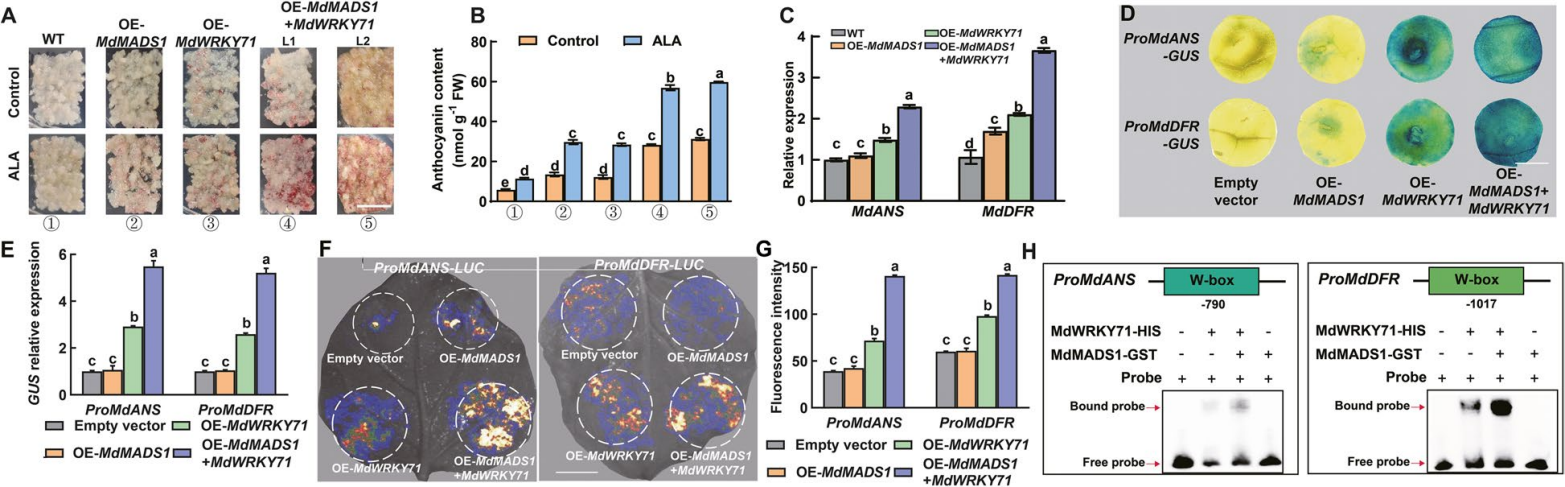
MdMADS1 promotes the transcriptional activation of MdWRKY71 on *MdANS* and *MdDFR*. **A** Coloration phenotype of two-week-old apple calli treated with ALA or without (Control) at 17 °C under 200 μmol m^−2^ s^−^^1^ light for half a month. Scale bar = 1 cm. **B** The anthocyanin content in (**A**). **C** Relative expression levels of *MdANS* and *MdDFR* in different transgenic apple calli. **D** GUS staining of tobacco leaves reveals that MdMADS1 itself cannot promote the activity of the promoters of *MdDFR* and *MdANS* but enhance the transcription regulatory activity of MdWRKY71 on the gene promoters. Scale bar = 1 cm. **E** RT-qPCR analysis confirms the relative *GUS* expression levels from the stained tobacco leaves in (**D**). **F** The luciferase reporter assays in tobacco leaves further demonstrate that MdMADS1 enhances the transcriptional activation of MdWRKY71 on *MdANS* and *MdDFR*. Scale bar = 2 cm. **G** Quantitative measurement of LUC fluorescence intensity as depicted in (**F**). **H** EMSA shows MdMADS1 increasing the binding strength of MdWRKY71 to W-box elements on *MdANS* and *MdDFR* promoters. Data are means ± SE of three biological replicates. Different lowercase letters in each panel represent significant differences *P* = 0.05

In order to verify the synergistic effect of MdMADS1 on MdWRKY71, we embarked on a stable co-expression analysis in apple calli (Figure S10). Our findings revealed that the co-transformation of OE-*MdWRKY71* and OE-*MdMADS1* resulted in a more pronounced accumulation of anthocyanins compared to the overexpression of a single gene (Fig. 8A, B). Notably, the anthocyanin levels were further elevated following the application of exogenous ALA, underscoring that ALA exerts the synergistic impact on the MdWRKY71-MdMADS1 interaction and the pigment biosynthesis.

Building upon our previous observations that MdWRKY71 enhances the binding of MdMADS1 to its promoters of target genes *MdCHS* and *MdUFGT* in Fig. 5, we sought to determine whether this synergistic interaction extends to the downstream genes, specifically *MdANS* and *MdDFR*. For this purpose, we measured the relative expression of *MdANS* and *MdDFR* in transgenic ‘Orin’ calli. The results showed that co-expression of OE-*MdWRKY71* and OE-*MdMADS1* further increased the expression levels of *MdANS* and *MdDFR* compared to single transformation of OE-*MdMADS1* or OE-MdWRKY71 (Fig. 8C). Next, we conducted *LUC* and *GUS* reporter gene assays. The results from the GUS assay indicated a more intense color development in tobacco leaves co-transformed with OE-*MdWRKY71* and OE-*MdMADS1* for both *MdANS* and *MdDFR* promoters, as opposed to those transformed with a single gene (Fig. 8D, E). Paralleling these findings, the LUC assay showed that although OE-*MdWRKY71* alone can activate the promoters of *MdANS* and *MdDFR*, the co-expression with *MdMADS1* significantly amplified this activation (Fig. 8F, G). Further corroborating these interactions, EMSA experiments demonstrated that the presence of MdMADS1 significantly bolstered the binding affinity of MdWRKY71-HIS to the promoters of *MdDFR* and *MdANS* (Fig. 8G). Collectively, these results suggest that the reciprocal interaction between MdWRKY71 and MdMADS1 augments the binding of MdWRKY71 to its downstream gene promoters, thereby enhancing gene expression and anthocyanin biosynthesis.

## Discussion

Given the importance of anthocyanins for high-quality fruit production, studies on the methods to improve anthocyanin accumulation and the regulatory mechanisms have been paid a lot of attention (Gao et al. 2021). ALA, a novel natural plant growth regulator, can promote fruit coloration outstandingly, showing great application potential in modern fruit production (Wang et al. 2004, 2023; Xie et al. 2013). Feng et al. (2016) previously found MdMADS1, a MADS-box TF, involved in ALA-improved anthocyanin accumulation in apple, but the mechanism is unclear. Here, we systematically deciphered the molecular mechanism of ALA-regulated anthocyanin biosynthesis in apple and revealed a new regulation module, the MdWRKY71-MdMADS1 module, for anthocyanin biosynthesis, different from the already well-known ternary MBW regulation complex (Zheng et al. 2021).

MADS-box TFs are integral to numerous fruit development processes in plants, including fruit ripening (Li et al. 2017; Cheng et al. 2019) and flavonoid biosynthesis (Nesi et al. 2002; Lalusin et al. 2006). Several studies have implicated MADS-box TFs in the regulation of anthocyanin production in plants. For example, in sweet potato, the *IbMADS10* expression pattern is closely correlated with anthocyanin levels in red roots, with overexpression in callus tissues leading to increased anthocyanin production (Lalusin et al. 2006). Similarly, in pears, the expression of *PyMADS18* during early fruit development is highly correlated with anthocyanin content (Wu et al. 2013). In strawberries, suppression of *FaMADS9* impacts normal fruit ripening and anthocyanin biosynthesis (Seymour et al. 2011). In apples, MdMADS1 plays a pivotal role in floral organ development and fruit ripening (Ireland et al. 2013), and our previous work has established its importance in ALA-enhanced apple coloration (Feng et al. 2016). Here, we further substantiate the positive regulatory function of MdMADS1 in ALA-mediated apple coloration enhancement through both transient leaf transformation and stable callus transformation experiments (Fig. 1). In contrast, ScAG and ScAGL11 in *Senecio cruentus* have been shown to reduce anthocyanin production in the ray florets (Qi et al. 2022), because they can bind to *CArG-*elements in the promoters of *ScF3H1* and *ScDFR3*, playing a negative regulatory role in anthocyanin accumulation. Bioinformatics analysis reveals that apple MdMADS1 is a canonical MADS-box TF. At least one CArG element, a potential binding site for MADS-box TFs, is found in the promoters of *MdCHS*, *MdANS*, *MdDFR*, and *MdUFGT*. Y1H, EMSA, and LUC assays demonstrated that MdMADS1 can directly bind to the *CArG* elements of *MdCHS* and *MdUFGT* but not *MdANS* and *MdDFR*, increasing the gene transcriptional activity (Fig. 2). These results conclusively demonstrate that MdMADS1 regulates anthocyanin biosynthesis by specifically identifying and binding to the promoters of structural genes in the anthocyanin biosynthetic pathway.

In plants, MYB, bHLH, and WD40 are known to form the MBW transcriptional activation complex, which activates one or more anthocyanin biosynthetic genes (Xu et al. 2015). In apples, the MBW members MYB1/10/A (Takos et al. 2006; Ban et al. 2007; Chagné et al. 2007; Espley et al. 2007, 2009; Hu et al. 2016), MdbHLH3/33 (Espley et al. 2007; Xie et al. 2012), and the WD40 protein TTG1 (An et al. 2012) have been characterized. Other TF types, such as B-box TFs, can also influence anthocyanin regulation by interacting with MBW components; for example, MdCOL11 interacts with MdMYBA in apple anthocyanin regulation (Bai et al. 2014). Similarly, AcMADS68 in red-fleshed kiwifruit interacts with AcMYBF110 and AcMYB123 to co-regulate anthocyanin biosynthesis (Liu et al. 2023). However, no interactions were found between MdMADS1 and the main reported MBW members in this study (Figure S5). Instead, we identified a WRKY TF, MdWRKY71, through Y2H screening using the full-length MdMADS1 protein as bait (Table S2). Both in vivo and in vitro experiments demonstrate the interaction between MdWRKY71 and MdMADS1 (Fig. 3), offering a novel avenue for further exploration distinct from the MBW regulatory pathway.

Several WRKY TFs in apple have been associated with anthocyanin biosynthesis, including MdWRKY1 (Ma et al. 2021), MdWRKY11 (Liu et al. 2019b), MdWRKY40 (An et al. 2019; Zhang et al. 2019), MdWRKY41 (Mao et al. 2021), MdWRKY71L (Su et al. 2022), and MdWRKY72 (Hu et al. 2020). In this study, we also validated the interaction between MdMADS1 and these WRKY TFs (Fig. 3A). However, only a specific interaction with MdWRKY71 was revealed. In addition, we demonstrated that MdWRKY71 contributes to ALA-regulated anthocyanin synthesis (Fig. 4) and that its interaction with MdMADS1 significantly enhances the binding of MdMADS1 to the promoters of *MdCHS* and *MdUFGT* (Fig. 5). These findings suggest that the MdMADS1-MdWRKY71 complex facilitates the expression of *MdCHS* and *MdUFGT* by enhancing the activity of MdMADS1.

WRKY TFs, as a major class of plant TFs, can directly bind to the promoters of target genes. For example, MdWRKY40 activates transcription by binding to the promoter of *MdANS* (Zhang et al. 2019), while MdWRKY11 promotes gene expression and anthocyanin biosynthesis by binding to the promoters of *MdUFGT* and several MYB TFs (Liu et al. 2019b). Similarly, MdWRKY71L enhances anthocyanin accumulation induced by ultraviolet-B by binding to the promoters of *MdMYB1* and *MdUFGT* (Su et al. 2022). In this study, we found that MdWRKY71 can bind to the *MdMADS1* promoter and upregulate its expression in Y1H, EMSA, LUC, and GUS reporter assays (Fig. 6). These results indicate that MdWRKY71 interacts with MdMADS1 at both the transcriptional and protein levels. Moreover, we discovered that MdWRKY71 can directly bind to the promoters of anthocyanin biosynthetic genes *MdANS* and *MdDFR*, thereby increasing their transcriptional activity (Fig. 7), suggesting an intrinsic regulatory role of MdWRKY71 in anthocyanin biosynthesis. Additionally, the co-expression of *MdMADS1* further enhanced the transcription activation activity of genes regulated by MdWRKY71 (Fig. 8). These results indicate that the MdMADS1-MdWRKY71 complex can also promote anthocyanin synthesis by enhancing the binding activity of MdWRKY71 to the target genes.

Additional to the findings in current study, our group has reported other transcriptional factor genes, such as *MdERF78* (Fang et al. 2022) *MdMYB110a* (Fang et al. 2023), and *MdNAC33* (Zhang et al. 2024), involved in ALA-induced anthocyanin biosynthesis*.* However, the possible regulatory factors more upstream beyond them in the ALA-induced apple coloration remain unclear. To understand the mechanism of action of ALA, we analyzed the promoter regions of these TFs and found that all TF genes possess multiple hormone-responsive elements as well as light-responsive elements in their upstream regions, with light-responsive elements being the most abundant (Figure S11). This leads us to hypothesize that light signaling may be a crucial factor in the ALA-induced anthocyanin accumulation, which provides a promising avenue for future investigative endeavors.

**Fig. 9:**
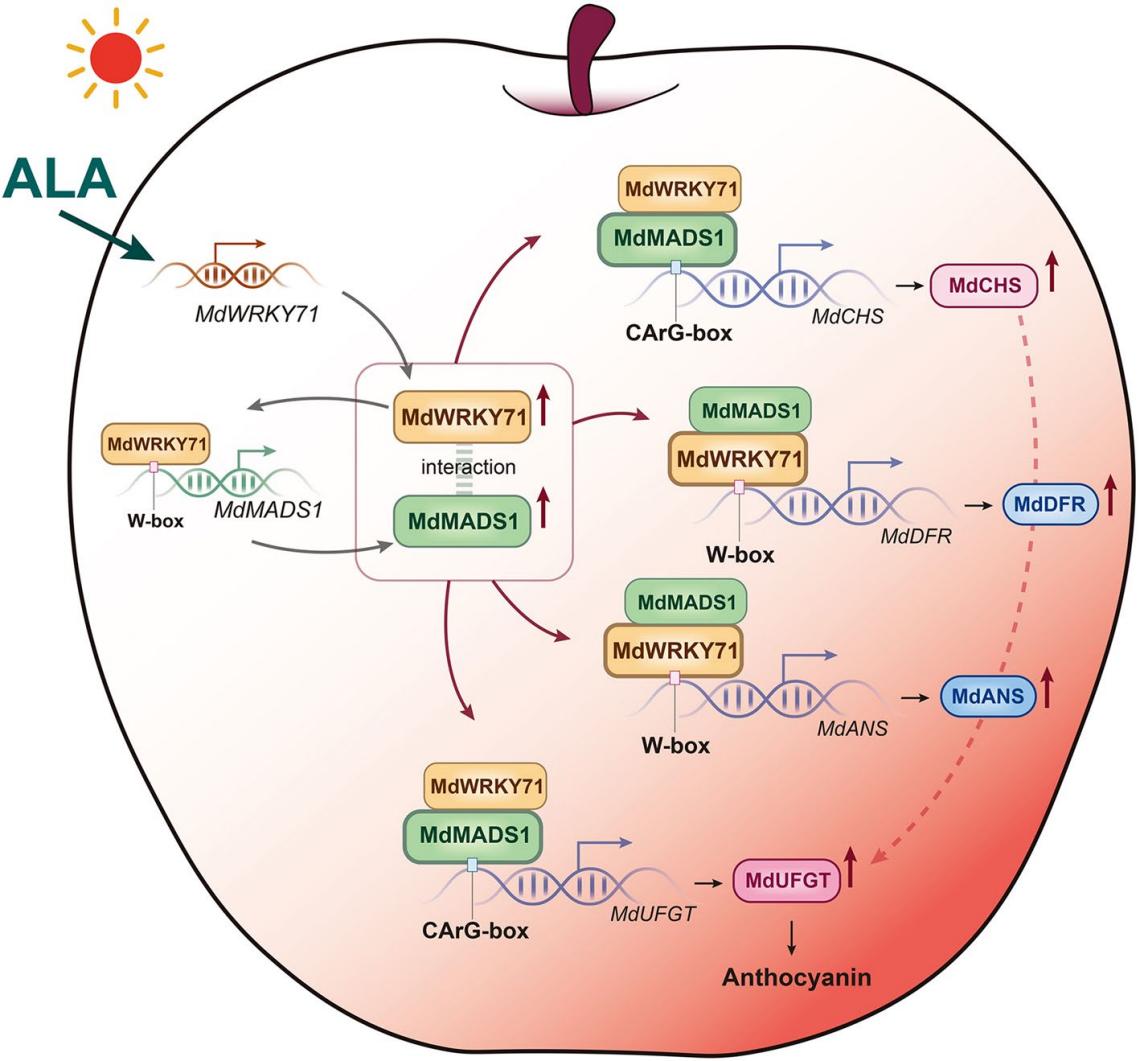
Model of transcriptional regulation of ALA-induced anthocyanin biosynthesis by the MdWRKY71-MdMADS1 module in apple skin. Upon ALA treatment, the transcription of *MdWRKY71* is activated, initiating a cascade of events. MdWRKY71 binds to the promoter of *MdMADS1* and activates the gene expression. Then, MdWRKY71 and MdMADS1, acting as a regulatory complex, independently or in one master and one auxiliary style modulate the expression of individual downstream genes in the anthocyanin biosynthetic pathway. MdWRKY71 directly binds with the W-boxes in the promoters of *MdANS* and *MdDFR*, enhancing the gene transcription. The transcriptional activation of MdWRKY71 can be amplified by interaction with MdMADS1. In parallel, MdMADS1 targets the CArG-boxes in the promoters of genes such as *MdCHS* and *MdUFGT*, further boosting their transcriptional activity in concert with MdWRKY71. In the cascade, MdWRKY71 is positioned upstream, exerting a positive regulatory influence on MdMADS1 at both the transcriptional activation and protein interaction levels. The synergistic complex of MdWRKY71 and MdMADS1 orchestrates the coordinated regulation of multiple downstream genes, collectively driving anthocyanin biosynthesis in the apple skin. This model elucidates an intricate interplay between two transcription factors in the ALA-induced enhancement of anthocyanin pigmentation

In conclusion, our findings reveal that both MdMADS1 and MdWRKY71 are integral to regulate ALA-induced anthocyanin biosynthesis. MdMADS1 directly activates the key biosynthetic genes *MdCHS* and *MdUFGT*, while MdWRKY71 activates *MdANS* and *MdDFR* expression. More importantly, MdWRKY71 enhances the MdMADS1 activity by binding the promoter to upregulate gene expression and interacting at the protein level to activate the transcriptional activity; while MdMADS1 promotes MdWRKY71 activity only by interacting with the protein. Therefore, in the regulation of *MdCHS* and *MdUFGT*, MdMADS1 plays a dominant role with assistance of MdWRKY71; conversely, for the expression of *MdANS* and *MdDFR*, MdWRKY71 takes the lead with supportive action from MdMADS1. Based on these findings, we have formulated a theoretical model that integrates both MdMADS1 and MdWRKY71 in the regulation of ALA-mediated anthocyanin accumulation (Fig. 9). These advance our understanding of the molecular mechanisms underlying ALA-induced anthocyanin production in plant tissues, highlighting the significance of the newly identified MdWRKY71-MdMADS1 module.

## Materials and methods

### Plant materials and treatments

Four cultivars of apple (*Malus* × *domestica* Borkh) were used in the study. Among them, the ‘Fuji’ calli were induced from the flesh, which were cultured or treated with exogenous ALA as described by Feng et al. (2016). ‘Orin’ calli, donated by Professor Chunxiang You for stable genetic transformation, were cultured in the dark and sub-cultured every two weeks (An et al. 2017). The ‘Royal Gala’ (GL-3) tube plantlets were grown under the conditions refer to Ren et al. (2021). Leaves of ‘GL-3’ were prepared for transient transformation. The ‘Huashuo’ fruits, harvested at the green mature stage in an orchard of Feng Xian County, Jiangsu Province, China, were utilized for both transient transformation and ALA treatment studies.

For ALA treatment, the ‘GL-3’ detached leaves were cut and pretreated by placing them on MS solid medium containing 25 μg L^−1^ ALA or no ALA and incubated in the dark for 12 h. The pretreated leaves were placed in an incubator at 17 °C and 200 μmol m^−2^ s^−1^ light intensity for continuous illumination. The ‘Fuji’ or ‘Orin’ calli were treated by 50 mg L^−1^ (Zheng et al. 2018) or 250 μg L^−1^ ALA (Zhang et al. 2024), respectively, which was added into the solid culture when calli were sub-cultured. When the fruits were treated by ALA, the detached ‘Huashuo’ apples were soaked in 200 mg L^−1^ ALA solution for 1–2 min, while the control apples were soaked in deionized water(Zhang et al. 2024). Then, all of them were kept in the dark at room temperature. After 24 h, they were transferred to a growth chamber at 17 °C with light intensity of 200 μmol m^−2^ s^−1^ for 72 h. Photographs were taken at 24, 48, 72 h, respectively. The samples of different time points were stored at − 80 °C.

### Anthocyanin extraction and determination

The anthocyanin content was quantified in accordance with Xie et al. (2012). To summarize concisely, the apple material was combined with 1 mL of a 1% (v/v) HCl-methanol solution and subjected to incubation at a temperature of 4 °C for a duration of 12 h in the dark. Then, the supernatant absorbance was measured at 530, 620, and 650 nm (Ubi et al. 2006), OD_λ_ = (A_530_ − A_620_) − 0.1 × (A_650_ − A_620_). Subsequently, the anthocyanin content was then quantified according to the formula: OD_λ_ / ξ_λ_ × V / m × 10^6^ (nmol/g; V, volume; m, weight; ξ_λ_: 3.43 × 10^4^).

### Total RNA extraction and real-time quantitative PCR analysis

The total RNA from the samples was extracted using the RNAprep Pure Plant Kit (DP441, TianGen, China) according to the manufacturer’s protocol. Complementary DNA (cDNA) was synthesized using the one-step gDNA Removal and cDNA Synthesis SuperMix (AT311-02, TransGen, China). Real-time quantitative PCR (RT-qPCR) was conducted using the SYBR Green Premix *Pro Taq* HS qPCR Kit (Rox Plus) (ACCURATE BIOTECHNOLOGY(HUNAN) CO., LTD, ChangSha, China).The relative expression of the genes was calculated using the 2^−△△CT^ method (Livak and Schmittgen 2001). The primers for RT-qPCR are listed in Supporting Information Table S1.

### Analysis of ALA on gene promoter activity

To investigate the promoter activity in response to ALA treatment, the 2000 bp promoter regions of *MdMADS1* and *MdWRKY71* were cloned into the pBI121-GUS vector to create the respective reporter constructs *ProMdMADS1-GUS* and *ProMdWRKY71-GUS*. These recombinant vectors were then transformed into *Agrobacterium tumefaciens* GV3101, which served as the delivery vehicle for transformation into various plant materials. The callus of ‘Orin’ apple was treated with 250 μg L^−1^ ALA, and the ‘GL-3’ leaves were subjected to 25 μg L^−1^ ALA for 3 days. Following the treatment, GUS histochemical staining was conducted to visualize and quantify the β-glucuronidase activity in these plant materials. The primer sequences utilized for cloning are listed in Table S1.

In addition, the *MdMADS1* promoter was constructed in the pGreenII0800-LUC plasmid to generate the *ProMdMADS1-LUC* constructs. The primers are listed in Table S1. The recombinant plasmid was transformed into *A. tumefaciens*GV3101 strain harboring the pSoup vector and injected into tobacco leaves (*Nicotiana benthamiana*). After a 12 h dark adaptation period at room temperature, the leaves were coated with 0.5 mg L^−1^ ALA (Chen et al. 2023). Following a 3-day incubation, an advanced imaging system (PIXIS 1024B, United States) was employed to detect and measure the LUC activity, providing a quantitative assessment of promoter responsiveness to ALA treatment.

### Cloning and sequence analysis of *MdMADS1* and *MdWRKY71*

The full-length CDS of *MdMADS1* and *MdWRKY71* were cloned from ‘Fuji’ apple. The primers are listed in Table S1. DNAMAN (Lynnon Biosoft, USA) was used to align the protein sequences. Phylogenetic analyses were conducted using the MEGA-X with the neighbor-joining method (Kumar et al. 2018). The amino acid sequences of MADS-box and WRKY transcription factors used for the phylogenetic tree construction are listed in Table S3 and Table S4, respectively.

### Transformation of apple calli, leaves and fruits

The coding sequences of *MdMADS1* and *MdWRKY71* were respectively cloned into pCAMBIA1300 vector to construct overexpression vectors OE-*MdMADS1* and OE-*MdWRKY71*. In order to build the RNA interference vector (RNAi), partial sequence (about 200–400 bp) of *MdMADS1* or *MdWRKY71* were respectively chosen to design specific primers and clone the interfering fragments, which were then recombined onto pHELLSGATE2 vector to construct RNAi-*MdMADS1* and RNAi-*MdWRKY71*. The primers are listed in Table S1. The recombinant vectors were transformed into *A. tumefaciens* LBA4404 and EHA105.

The ‘Orin’ calli were used for stable transformation as previously described (An et al. 2019), while ‘GL-3’ leaves were used for transient transformation, which was performed through vacuum infiltration according to the previous method (An et al. 2020). The infected leaves were cultured in darkness on the MS solid medium for 12 h before undergoing subsequent ALA treatment. For apple fruit transformation, EHA105 cells containing expression vectors were injected into the ‘Huashuo’ peel. Subsequently, the apple fruit was stored in the dark for 12 h and then incubated in a growth environment under constant light at 17 °C and 200 μmol m^−2^ s^−1^ light to induce coloring. All transgenic materials were identified by PCR and RT-qPCR (Figure S2; Figure S7; Figure S10).

### Yeast one-hybrid (Y1H) assays

The CDS of *MdMADS1* or *MdWRKY71* was recombined into the pGADT7 vector. The bait sequences of *ProMdCHS*, *ProMdF3H*, *ProMdDFR*, *ProMdANS*, *ProMdUFGT*, *ProMdGSTF12, ProMdMADS1, and ProMdWRKY71* were recombined into the pAbAi vector (Clontech, Beijing). The methods were performed according to Liu et al. (2019a). The primers for vector construction are listed in Table S1.

### Luciferase reporter (LUC) analysis

The 2000 bp promoter regions of *MdCHS*, *MdDFR*, *MdANS*, *MdUFGT,* and *MdMADS1* were recombined into pGreenII0800-LUC vector. The CDS of *MdMADS1* or *MdWRKY71* was cloned into pCAMBIA1300 vector to produce effector constructs. One-month-old tobacco leaves were used in transient transfection experiments. After infiltration, the LUC signal was observed and the LUC intensity was subsequently measured with ImageJ software. The primers for vector construction are listed in Table S1.

### Electrophoretic mobility shift assay (EMSA)

The *MdWRKY71* or *MdMADS1* CDS was separately inserted into the pET32a (6 × His) and pGEX4T-1 (GST) vectors. The primers are listed in Table S1. The MdWRKY71-His fusion protein was purified with the Kit (Cwbiotech, Beijing, China). The MdMADS1-GST recombinant protein was purified with the Kit (Beyotime Biotech, Shanghai, China). The probe was labeled with biotin by Shanghai Generay Biotech Co., Ltd. (Shanghai, China). The EMSA reactions were prepared according to the manufacturer's instructions (LightShift Chemiluminescent EMSA Kit; Thermo Fisher Scientific, Shanghai, China).

### Yeast two-hybrid (Y2H) screening and analysis

The CDS of *MdMYB10*, *MdbHLH3*, *MdbHLH33*, *MdTTG1*, *MdWRKY1*, *MdWRKY11*, *MdWRKY40*, *MdWRKY41*, *MdWRKY71L*, *MdWRKY72* and *MdWRKY71* were recombined into pGADT7-AD vector. The CDS of *MdMADS1* was inserted into pGBKT7-BD vector. The primers are listed in Table S1. The experiment was carried out in accordance with the protocols (Clontech, Beijing, China). To screen the proteins that interact with MdMADS1, Y2H screening was conducted following the instructions (Clontech, Beijing, China). One-on-one analysis was performed to confirm the interaction.

### Pull-down assay

For the in vitro pull-down experiment, the induced purified MdWRKY71-HIS protein was co-incubated with MdMADS1-GST protein or GST-tagged bait protein. After incubation, these proteins were eluted in the elution buffer. Finally, immunoblotting analysis was performed using anti-HIS and anti-GST antibodies (Beyotime Biotech, shanghai, China).

### Subcellular localization analysis

The CDS without stop codon for *MdMADS1* or *MdWRKY71* were inserted into pCAMBIA1300*-GFP* to generate *35S::MdMADS1-GFP* and *35S::MdWRKY71-GFP*. The primers are listed in Table S1. The tobacco leaves was infiltrated with pCAMBIA1300*-GFP*, *35S::MdMADS1-GFP* and *35S::MdWRKY71-GFP*, respectively. Confocal microscopy was employed to detect the GFP fluorescence signal (LSM 780, Zeiss, Germany).

### Bimolecular fluorescence complementation (BiFC) assay

The CDS without stop codon of *MdMADS1* was ligated into *pSPYNE* vector to generate *NYFP-MdMADS1*. And the CDS without stop codon of *MdMYB10, MdbHLH3, MdbHLH33, MdTTG1* and *MdWRKY71* were respectively inserted into *pSPYCE* vector to generate *MdMYB10-CYFP*, *MdbHLH3-CYFP*, *MdbHLH33-CYFP*, *MdTTG1-CYFP* and *MdWRKY71-CYFP*. The primers for vector construction are listed in Table S1. The tobacco leaves were used to co-infiltrate the recombinant plasmids. Confocal microscopy was employed to detect the fluorescence signal (LSM 780, Zeiss, Germany).

### Luciferase complementation imaging (LCI) assay

LCI assay was carried out as previously described by Chen et al. (2008). The tobacco leaves were used to co-infiltrate the recombinant plasmids pCAMBIA1300*-MdMADS1-nLUC* and pCAMBIA1300*-cLUC-MdWRKY71*. The primers for vector construction are listed in Table S1. An imaging system was employed to monitor the fluorescence signals (PIXIS 1024B, United States).

### GUS staining analysis

The 2000 bp promoter regions for *MdMADS1*, *MdWRKY71*, *MdCHS*, *MdANS*, *MdDFR,* and *MdUFGT* were fused with pBI121-GUS to replace the CaMV35S as the reporter vectors. The full-length CDS of *MdMADS1* and *MdWRKY71* were combined into a vector driven by CaMV35S as effector vectors. The primers are listed in Table S1. GUS staining analysis was performed as previously described (Franco-Zorrilla et al. 2007). In short, plant materials were placed in GUS staining buffer (1 mM X-Gluc, 0.5 mM ferrocyanide, 0.5 mM ferricyanide, 0.1% Triton X-100, and 0.1 mM EDTA) for staining, followed by destaining with absolute ethanol.

### Statistical analysis

All experiments were performed at least three biological replications. SPSS 20.0 was used to carry out a statistical analysis that included variance and significant differences. A significant difference will be assessed to exist when the *P*-value was less than 0.05.

## Supplementary Information

Additional file 1: Figure S1. ALA treatment induced anthocyanin accumulation in ‘Fuji’ apple calli. Figure S2. Identification of *MdMADS1*-transformed ‘Orin’ apple calli by PCR and RT-qPCR. Figure S3. Multiple sequence alignment and structure analysis of the MdMADS1. Figure S4. No interaction of MdMADS1 with promoters of anthocyanin biosynthetic and transport genes analyzed by Y1H. Figure S5. No interaction between MdMADS1 and MdMYB10, MdbHLH3, MdbHLH33 or MdTTG1 analyzed by Y2H and BiFC. Figure S6. Multiple sequence alignment and structure analysis of MdWRKY71. Figure S7. Identification of *MdWRKY71*-transformed ‘Orin’ apple calli by PCR and RT-qPCR. Figure S8. MdMADS1 cannot bind to the promoter of MdWRKY71 in Y1H and GUS staining. Figure S9. Y1H analysis of the interaction of MdWRKY71 with promoters of anthocyanin biosynthetic and transport genes. Figure S10. Identification of *MdWRKY71*-*MdMADS1* transformed ‘Orin’ apple calli by PCR and RT-qPCR. Figure S11.Analysis of *cis*-acting elements in the promoters of *MdERF78*, *MdMYB110a*, *MdNAC33*, *MdMADS1*, and *MdWRKY71*.

Additional file 2:Table S1. All primer sequences synthesized and used in this study. Table S2. Results of yeast two-hybrid library screening using MdMADS1 as a bait. Table S3. The protein sequences of MADS-box TFs from different species used to construct phylogenetic tree. Table S4. The protein sequences of WRKY-TFs from different species used to construct phylogenetic tree.

## Data Availability

Data will be available from the corresponding author upon reasonable request.

## Abbreviations

LCI: Luciferase complementation imaging
GFP: Green fluorescent protein
LUC: Firefly luciferase
CDS: Coding sequence
RT-qPCR: Quantitative real-time polymerase chain reaction
TFs: Transcription factors
Y1H: Yeast one-hybrid assay
Y2H: Yeast two-hybrid assay
EMSA: Electrophoretic mobility shift assay
