# Supplementary material for: 5-Aminolevulinic acid activates the MdWRKY71-MdMADS1 module to enhance anthocyanin biosynthesis in apple
Source: Mol Hortic. 2025 Feb 3;5:10. doi: 10.1186/s43897-024-00127-x (PMC11789342; doi:10.1186/s43897-024-00127-x)
Supplement: Supplementary file 1 — Additional file 1: Figure S1. ALA treatment induced anthocyanin accumulation in ‘Fuji’ apple calli. Figure S2. Identification of MdMADS1-transformed ‘Orin’ apple calli by PCR and RT-qPCR. Figure S3. Multiple sequence alignment and structure analysis of the MdMADS1. Figure S4. No interaction of MdMADS1 with promoters of anthocyanin biosynthetic and transport genes analyzed by Y1H. Figure S5. No interaction between MdMADS1 and MdMYB10, MdbHLH3, MdbHLH33 or MdTTG1 analyzed by Y2H and BiFC. Figure S6. Multiple sequence alignment and structure analysis of MdWRKY71. Figure S7. Identification of MdWRKY71-transformed ‘Orin’ apple calli by PCR and RT-qPCR. Figure S8. MdMADS1 cannot bind to the promoter of MdWRKY71 in Y1H and GUS staining. Figure S9. Y1H analysis of the interaction of MdWRKY71 with promoters of anthocyanin biosynthetic and transport genes. Figure S10. Identification of MdWRKY71-MdMADS1 transformed ‘Orin’ apple calli by PCR and RT-qPCR. Figure S11.Analysis of cis-acting elements in the promoters of MdERF78, MdMYB110a, MdNAC33, MdMADS1, and MdWRKY71. [file 43897_2024_127_MOESM1_ESM.docx]

**Supplementary Figure**

**Article title: 5-Aminolevulinic acid activates the MdWRKY71-MdMADS1 module to enhance anthocyanin accumulation in apple**

**Authors:** **Liuzi Zhang^a^, Huihui Tao^a^, Jianting Zhang^a^, Yuyan An^b^*, Liangju Wang^a^***

**^a^College of Horticulture, Nanjing Agricultural University, Nanjing 210095, China**

**^b^College of Life Sciences, Shaanxi Normal University, Xi’an 710119, China**

**Figure S1**. ALA treatment induced anthocyanin accumulation in ‘Fuji’ apple calli.

**Figure S2**. Identification of *MdMADS1*-transformed ‘Orin’ apple calli by PCR and RT-qPCR.

**Figure S3**. Multiple sequence alignment and structure analysis of the MdMADS1.

**Figure S4**. No interaction of MdMADS1 with promoters of anthocyanin biosynthetic and transport genes analyzed by Y1H.

**Figure S5**. No interaction between MdMADS1 and MdMYB10, MdbHLH3, MdbHLH33 or MdTTG1 analyzed by Y2H and BiFC.

**Figure S6**. Multiple sequence alignment and structure analysis of MdWRKY71.

**Figure S7**. Identification of *MdWRKY71*-transformed ‘Orin’ apple calli by PCR and RT-qPCR.

**Figure S8**. MdMADS1 cannot bind to the promoter of MdWRKY71 in Y1H and GUS staining.

**Figure S9**. Y1H analysis of the interaction of MdWRKY71 with promoters of anthocyanin biosynthetic and transport genes.

**Figure S10**. Identification of *MdWRKY71*-*MdMADS1* transformed ‘Orin’ apple calli by PCR and RT-qPCR.

**Figure S11**.Analysis of *cis*-acting elements in the promoters of *MdERF78*, *MdMYB110a*, *MdNAC33*, *MdMADS1*, and *MdWRKY71*.


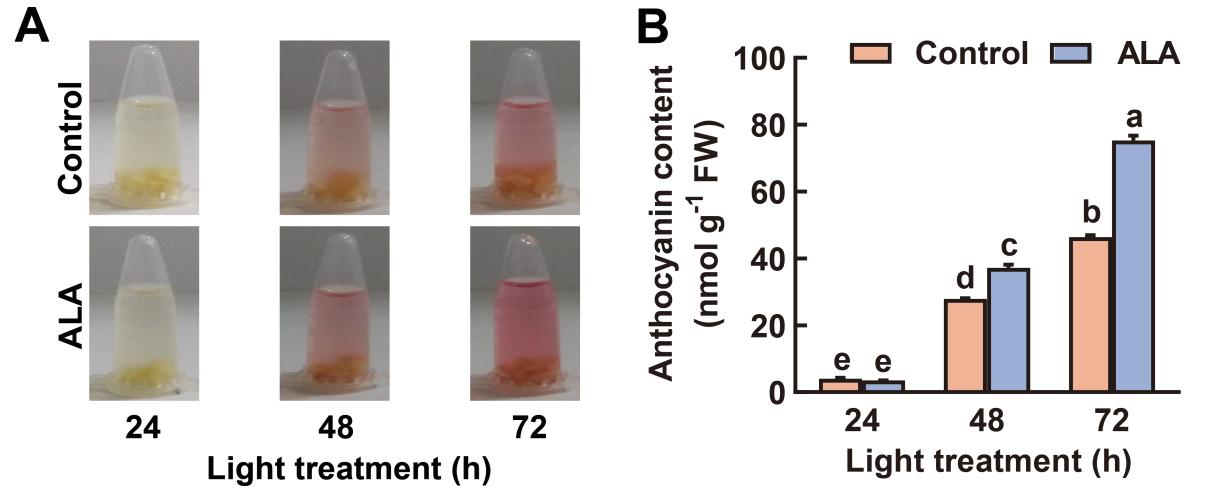


**Fig S1. ALA treatment induced anthocyanin accumulation in ‘Fuji’ apple calli.**

(**A**) The 'Fuji' apple calli accumulates more anthocyanins when treated with ALA. The calli were cultured on MS solid media added with 50 mg L^-1^ ALA or without (control) in dark for 12 h, then transferred to the growth chamber of 200 µmol m^−2^ s^−1^ constant light at 17°C for 72 h. (**B**) The callus anthocyanin content in (**A**). The data in the figure are means ± SE of three biological replicates. The different small letters in each panel represent significant differences *P* = 0.05.


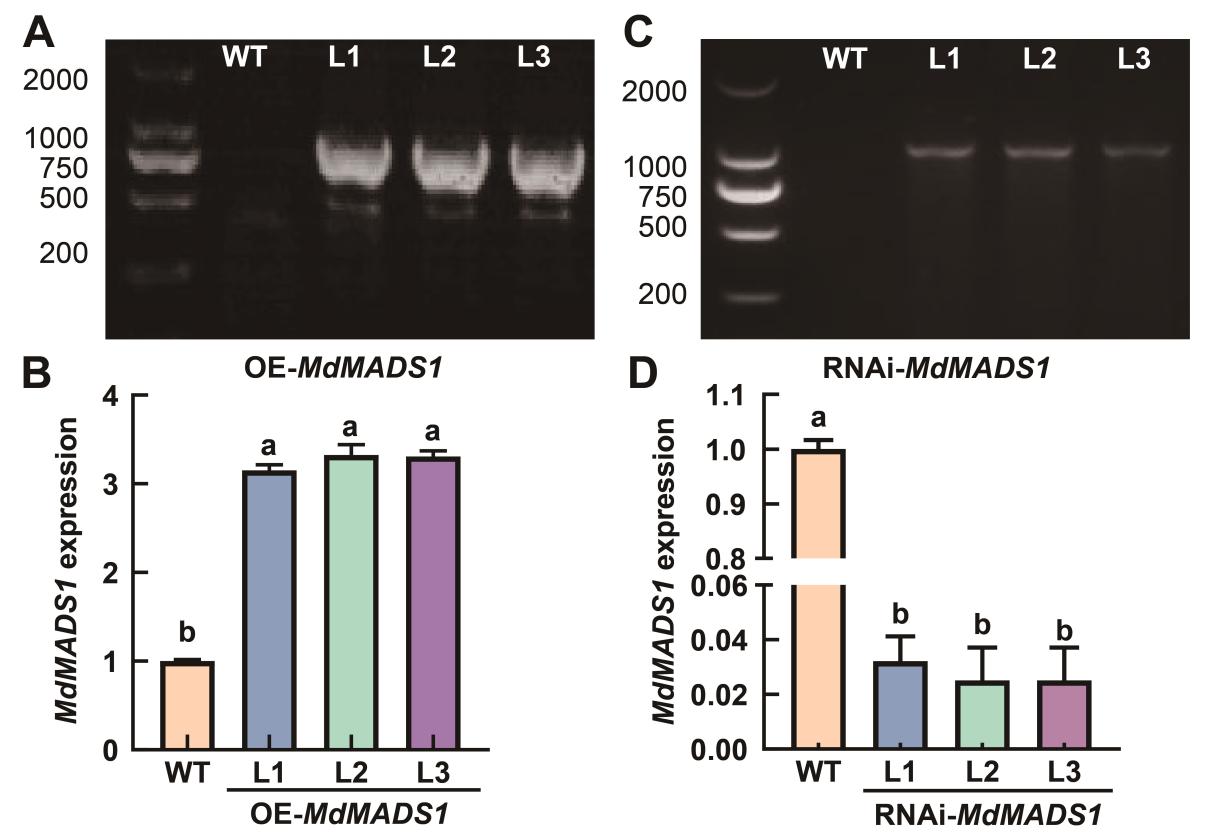


**Fig S2. Identification of *MdMADS1*-transformed ‘Orin’ apple calli by PCR and RT-qPCR.**

(**A** and **C)** Confirmation of transgenic lines by PCR amplification. (**B** and **D**) The expressions of *MdMADS1* in different transgenic lines were determined by RT-qPCR. The data in the figure are means ± SE of three biological replicates. The different small letters in each panel represent significant differences *P* = 0.05.

**
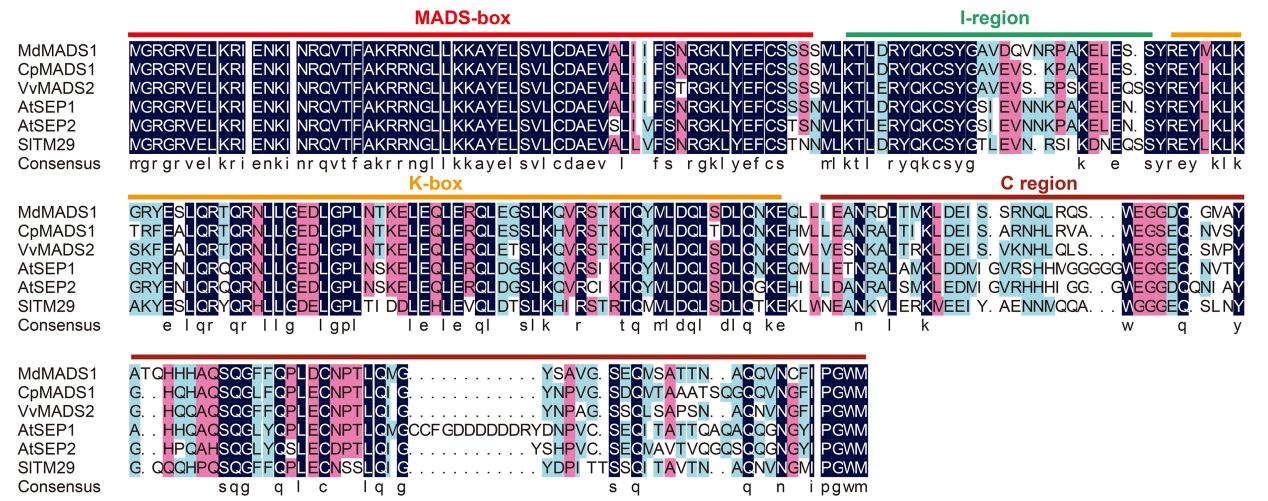
**

**Fig S3. Multiple sequence alignment and structure analysis of the MdMADS1.**

The protein sequences of MdMADS1 (*Malus x domestica*), CpMADS1 (*Carica papaya*), VvMADS2 (*Vitis vinifera*), AtSEP1 (*Arabidopsis thaliana*), AtSEP2 (*Arabidopsis thaliana*), and SlTM29 (*Solanum lycopersicum*) are aligned. The red and orange lines above the alignment localize the MADS-box and K-box structural domains, respectively. The green and brown lines above the alignment localize the I region and C regions.

**
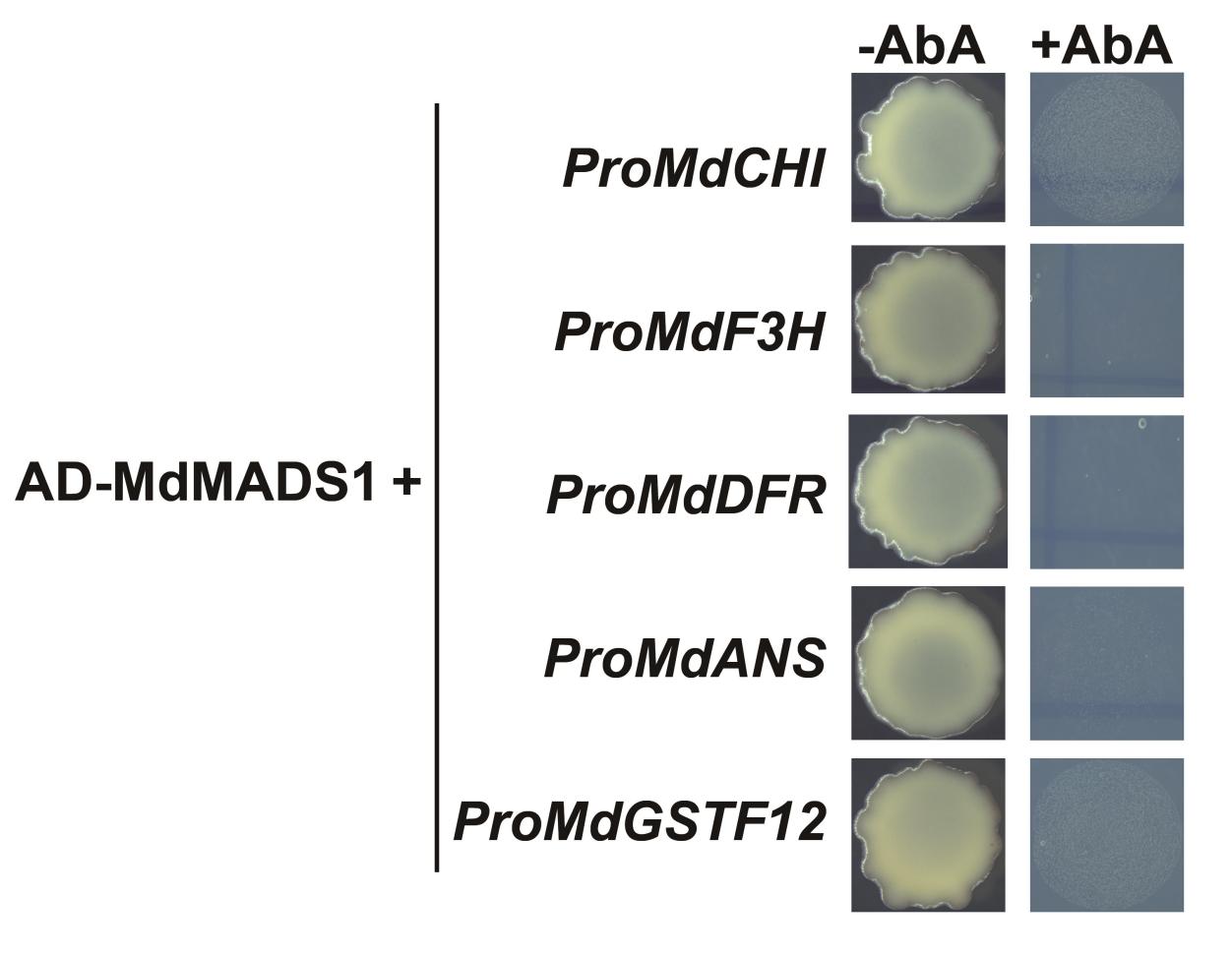
**

**Fig S4. No interaction of MdMADS1 with promoters of anthocyanin biosynthetic and transport genes analyzed by Y1H.**

**
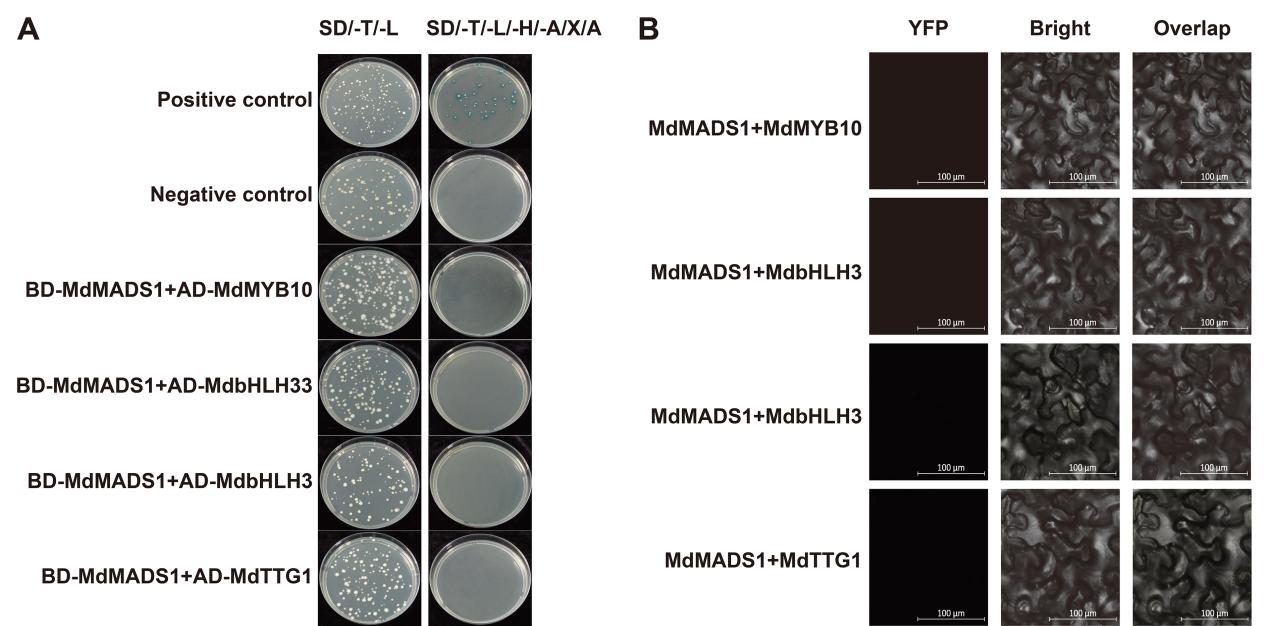
Fig S5. No interaction between MdMADS1 and MdMYB10, MdbHLH3, MdbHLH33 or MdTTG1 analyzed by Y2H and BiFC.**

(**A**) Y2H assay to examine any interaction between MdMADS1 and MdMYB10, MdbHLH3, MdbHLH33 and MdTTG1, respectively. Yeast cells were grown on SD/-Trp-Leu (-T/-L) and SD/-Trp-Leu-His-Ade/X-α-gal/AbA (-T/-L/-H/-A/X/AbA) media. (**B**) BiFC assay was used to examine any interaction between MdMADS1 and MBW components detected by transiently transformed *N. benthamiana* leaf epidermis. YFP fluorescence was observed via confocal microscopy. Scale bar = 100 μm.

**
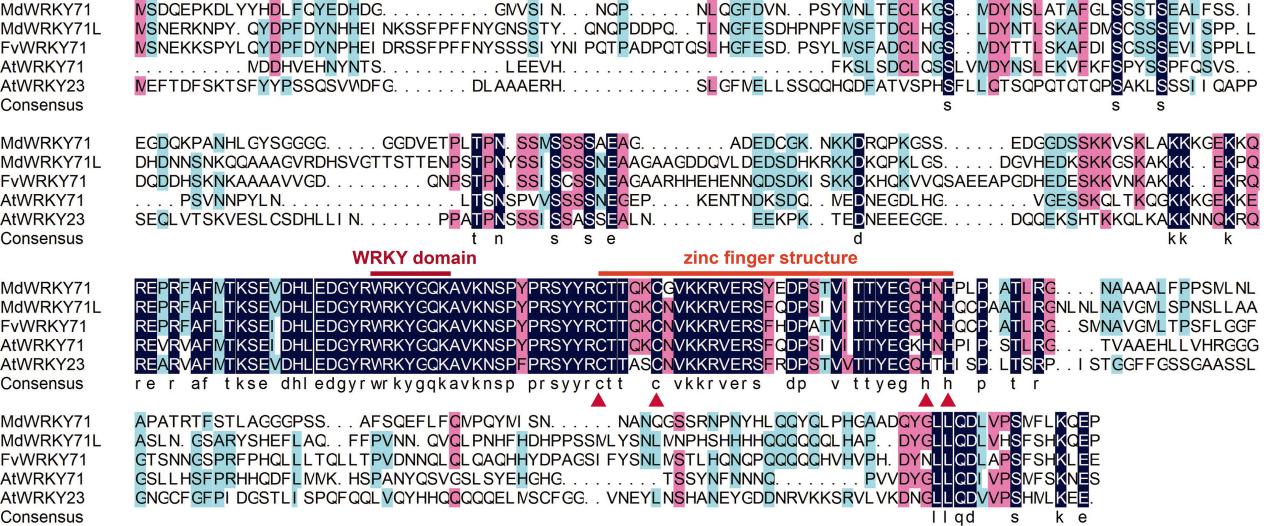
Fig S6. Multiple sequence alignment and structure analysis of MdWRKY71.**

The protein sequences of MdWRKY71 (*Malus* x *domestica*), MdWRKY71L (*Malus* x *domestica*), FvWRKY71 (*Fragaria vesca*), AtWRKY71 (*Arabidopsis thaliana*), and AtWRKY23 (*Arabidopsis thaliana*) are aligned here. The purple line above the alignment locates the WRKY structural domain. The red line above the alignment indicates the position of the zinc finger motif.

**
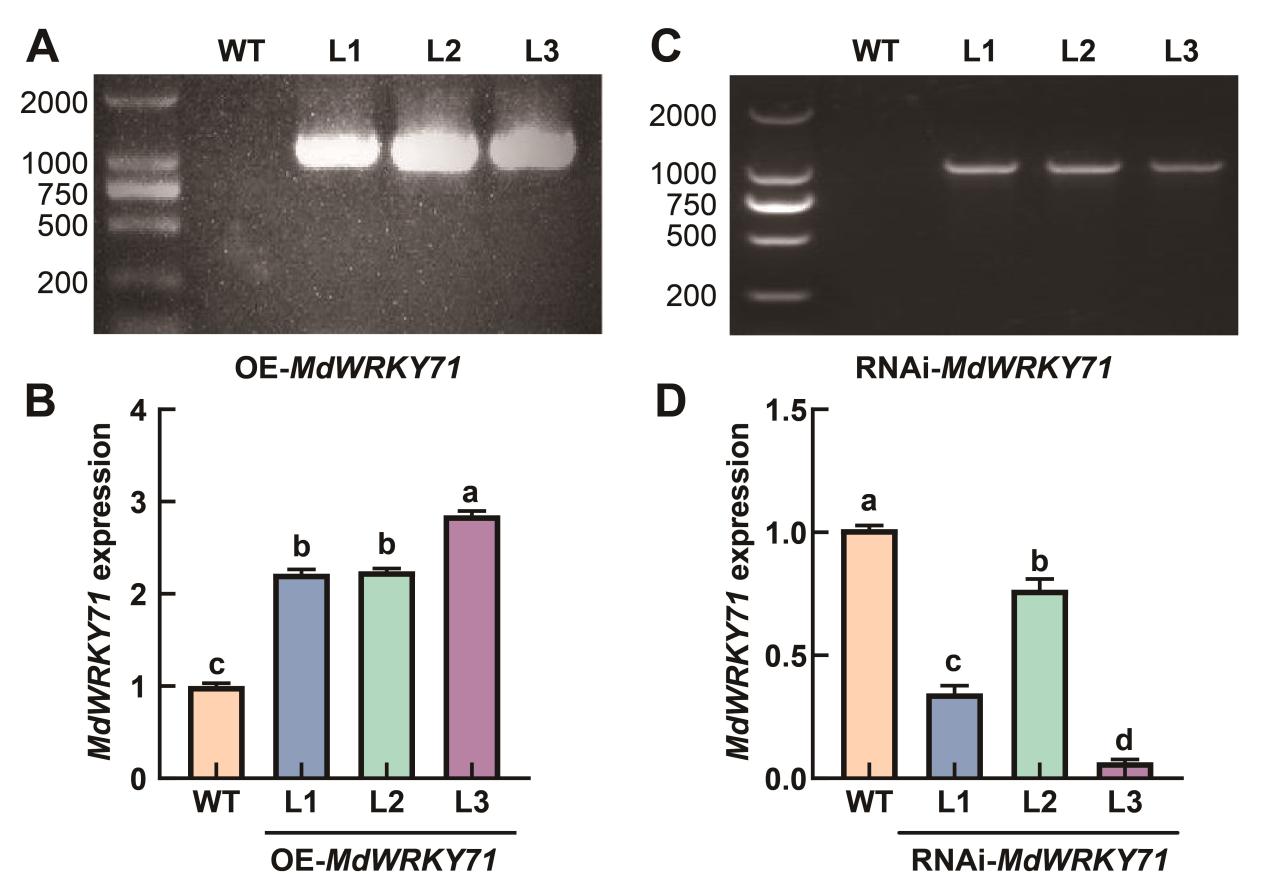
Fig S7. Identification of *MdWRKY71*-transformed ‘Orin’ apple calli by PCR and RT-qPCR.**

(**A** and **C)** Confirmation of transgenic lines by PCR amplification. (**B** and **D**) The expressions of *MdWRKY71* in different transgenic lines were determined by RT-qPCR. The data in the figure are means ± SE of three biological replicates. The different small letters in each panel represent significant differences *P* = 0.05.

**
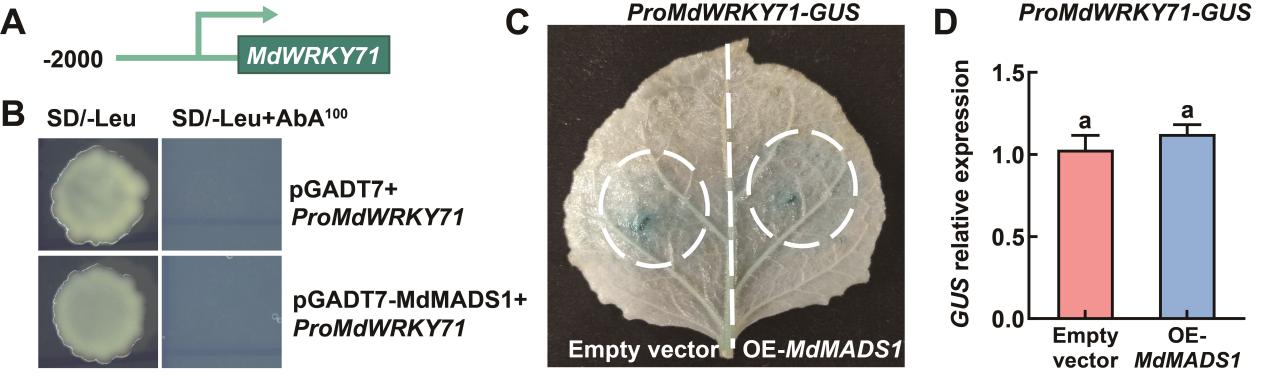
Fig S8. MdMADS1 cannot bind to the promoter of MdWRKY71 in Y1H and GUS staining.**

(**A**) Schematic diagram of *MdWRKY71* promoters. (**B**) Y1H assays show no interaction between MdMADS1 and the promoter of *MdWRKY71*. (**C-D**) Transient transactivation assay using the *GUS* reporter gene. GUS staining in leaves co-transformed with the *MdMADS1* and *MdWRKY71* promoter. Each experiment was performed in three replicates. Error bars denote standard errors. The same letters above the bars indicate no significant difference at *P* = 0.05.

**
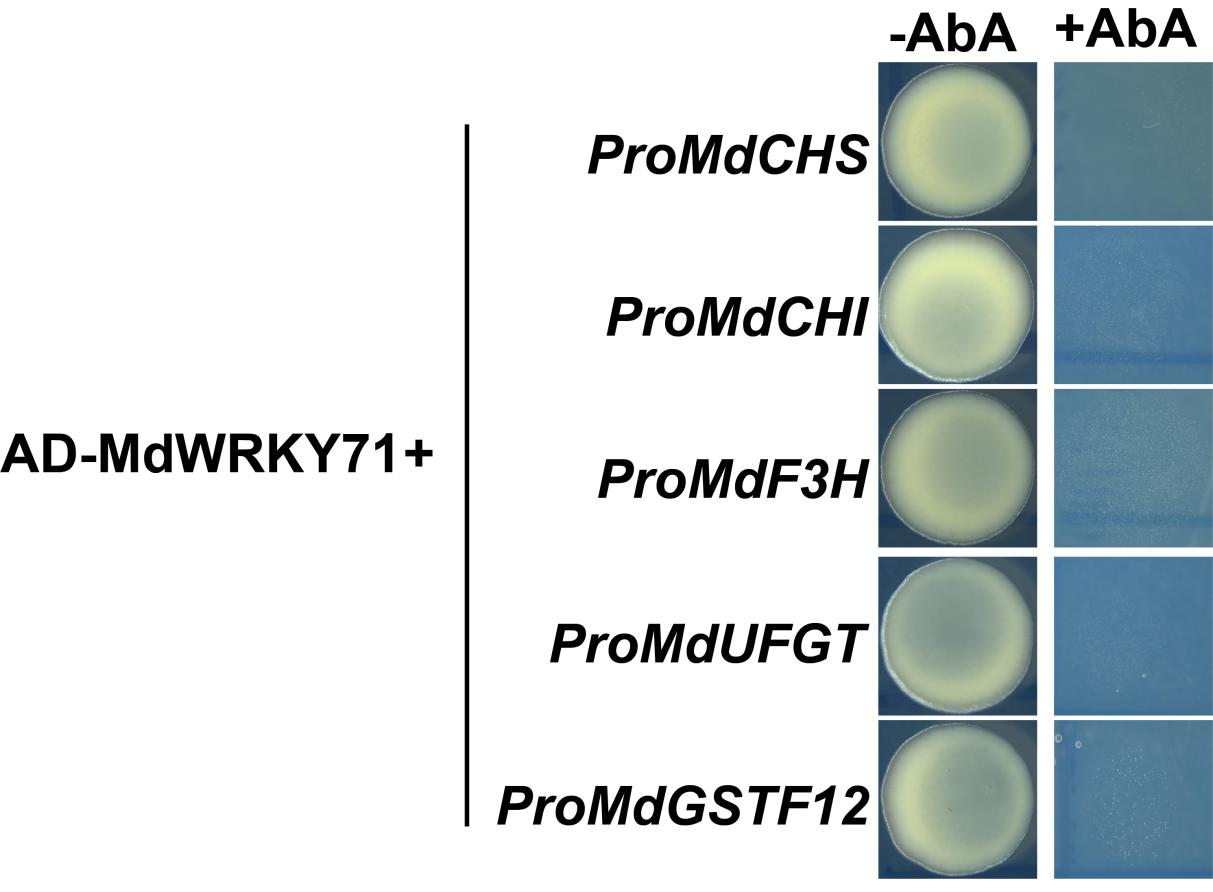
**

**Fig S9. Y1H analysis of the interaction of MdWRKY71 with promoters of anthocyanin biosynthetic and transport genes.**

**
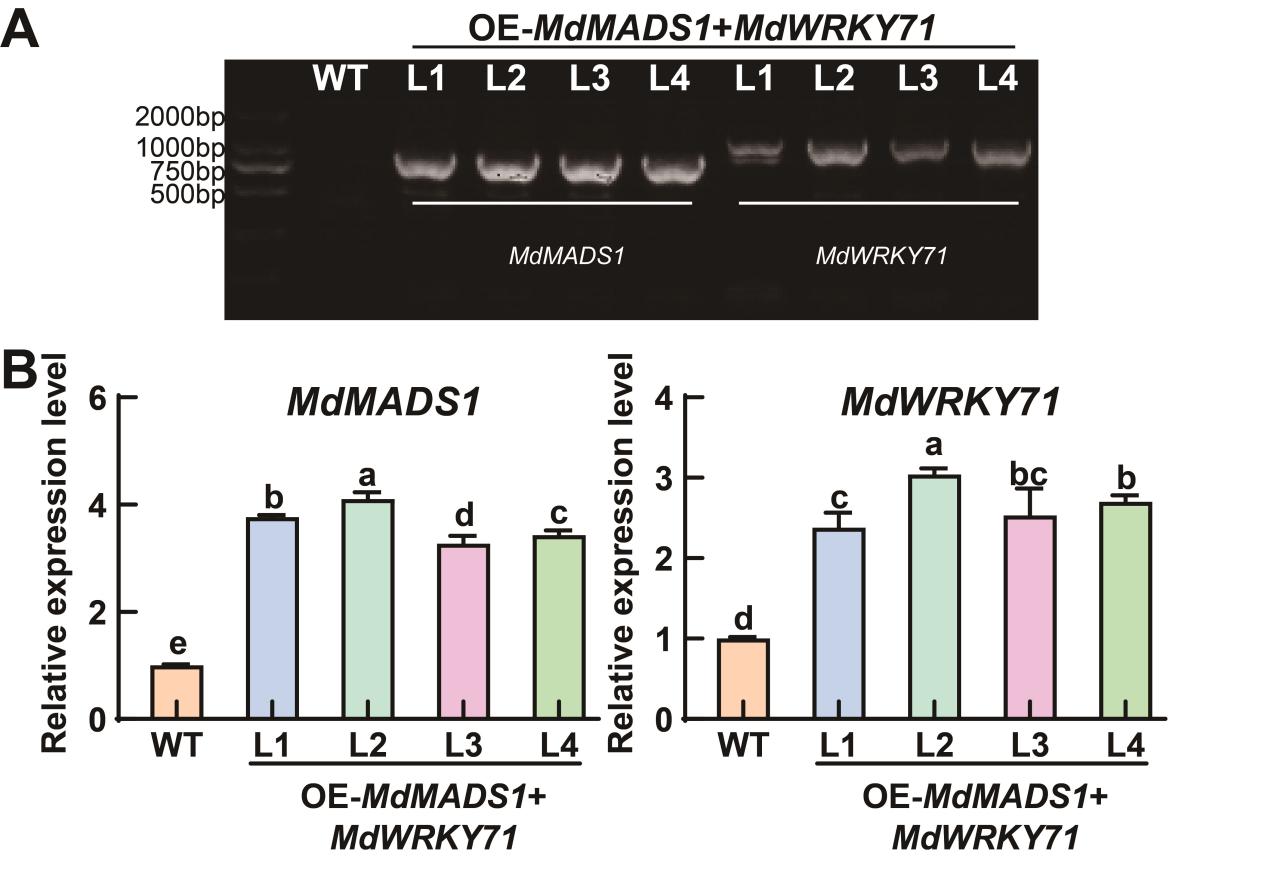
Fig S10. Identification of *MdWRKY71*-*MdMADS1* transformed ‘Orin’ apple calli by PCR and RT-qPCR.**

(**A)** Confirmation of transgenic lines by PCR amplification. (**B**) The expressions of *MdWRKY71* and *MdMADS1* in different transgenic lines were determined by RT-qPCR. The data in the figure are means ± SE of three biological replicates. The different small letters in each panel represent significant differences *P* = 0.05.

**
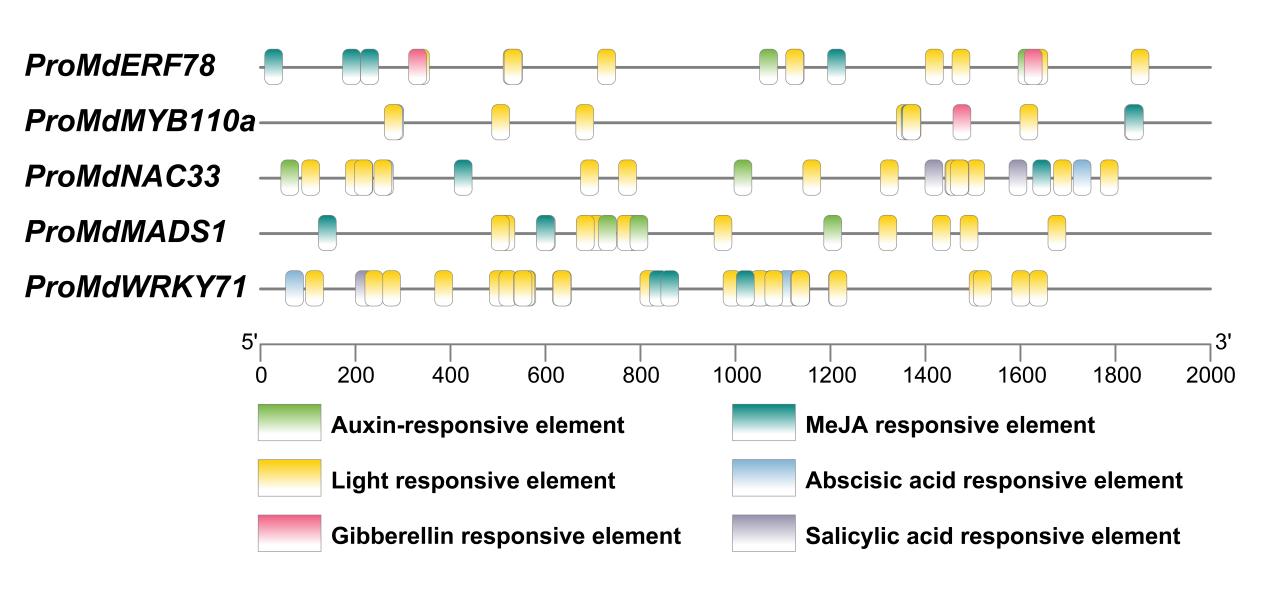
Fig S11.Analysis of *cis*-acting elements in the promoters of *MdERF78*, *MdMYB110a*, *MdNAC33*, *MdMADS1*, and *MdWRKY71*.**

Perform cis-acting element analysis by extracting the 2000bp upstream sequence of the start codon ATG of these genes. Visualize the results using TBtools software.
